# Heterologous Gln/Asn-Rich Proteins Impede the Propagation of Yeast Prions by Altering Chaperone Availability

**DOI:** 10.1371/journal.pgen.1003236

**Published:** 2013-01-24

**Authors:** Zi Yang, Joo Y. Hong, Irina L. Derkatch, Susan W. Liebman

**Affiliations:** 1 Department of Biological Sciences, University of Illinois at Chicago, Chicago, Illinois, United States of America; 2 Department of Biochemistry and Molecular Biology, University of Nevada, Reno, Nevada, United States of America; 3 Department of Neuroscience, Columbia University, New York, New York, United States of America; The University of Arizona, United States of America

## Abstract

Prions are self-propagating conformations of proteins that can cause heritable phenotypic traits. Most yeast prions contain glutamine (Q)/asparagine (N)-rich domains that facilitate the accumulation of the protein into amyloid-like aggregates. Efficient transmission of these infectious aggregates to daughter cells requires that chaperones, including Hsp104 and Sis1, continually sever the aggregates into smaller “seeds.” We previously identified 11 proteins with Q/N-rich domains that, when overproduced, facilitate the *de novo* aggregation of the Sup35 protein into the [*PSI* ^+^] prion state. Here, we show that overexpression of many of the same 11 Q/N-rich proteins can also destabilize pre-existing [*PSI* ^+^] or [*URE3*] prions. We explore in detail the events leading to the loss (curing) of [*PSI^+^*] by the overexpression of one of these proteins, the Q/N-rich domain of Pin4, which causes Sup35 aggregates to increase in size and decrease in transmissibility to daughter cells. We show that the Pin4 Q/N-rich domain sequesters Hsp104 and Sis1 chaperones away from the diffuse cytoplasmic pool. Thus, a mechanism by which heterologous Q/N-rich proteins impair prion propagation appears to be the loss of cytoplasmic Hsp104 and Sis1 available to sever [*PSI* ^+^].

## Author Summary

Certain proteins can occasionally misfold into infectious aggregates called prions. Once formed, these aggregates grow by attracting the soluble form of that protein to join them. The presence of these aggregates can cause profound effects on cells and, in humans, can cause diseases such as transmissible spongiform encephalopathies (TSEs). In yeast, the aggregates are efficiently transmitted to daughter cells because they are cut into small pieces by molecular scissors (chaperones). Here we show that heritable prion aggregates are frequently lost when we overproduce certain other proteins with curing activity. We analyzed one such protein in detail and found that when it is overproduced it forms aggregates that sequester chaperones. This sequestration appears to block the ability of the chaperones to cut the prion aggregates. The result is that the prions get too large to be transmitted to daughter cells. Such sequestration of molecular scissors provides a potential approach to thwart the propagation of disease-causing infectious protein aggregates.

## Introduction

The infectivity of transmissible spongiform encephalopathies (TSEs) was explained by the prion hypothesis proposing that the inheritance of biological information can be achieved by self-propagating conformational changes in the prion protein PrP [1]. The prion list has since been extended to include protein-based genetic elements found in fungi [2]. The best-studied yeast prions [*PSI* ^+^], [*PIN* ^+^] (often called [*RNQ* ^+^]) and [*URE3*] are, respectively, self-propagating conformations of: Sup35, a translation termination factor; Rnq1, a protein of unknown function; and Ure2, a nitrogen catabolism repression regulator [3]–[5]. Other recently discovered yeast prions include [*SWI* ^+^], [*OCT* ^+^], [*ISP* ^+^], [*MOT3* ^+^] and [*MOD5*] [6], [7]. The propagation of most [8]–[10], but not all [6] yeast prions is driven by their Q/N-rich prion domains that have the propensity to form aggregates *in vivo* and assemble into self-seeding, β-sheet-rich amyloid fibers *in vitro* 11,12.

Prion propagation involves templated conversion of soluble protein into the prion state [13] . *In vitro* data show that amyloid fibers grow by recruiting protein monomers to fiber ends [14]. In addition, prion propagation requires fibers to be fragmented to create new ends for conversion and to allow efficient transmission of seeds to daughter cells [15]. Failure at any of these steps would lead to loss (curing) of the prion.

The Hsp104 chaperone is required for the propagation of yeast prions, and its elimination leads to prion loss [16], [17]. One role of Hsp104 in prion propagation is to shear prion aggregates [16], [18]–[23]. Overexpression of Hsp104 also cures cells of [*PSI* ^+^] [16]. The mechanism appears to be more complex than simple over-shearing [24]–[28]. A recent study indicates that Hsp104 overexpression displaces the Hsp70 chaperone Ssa1 from binding to [*PSI* ^+^] aggregates. Since Ssa1 is required for Hsp104 shearing activity, this inhibits shearing of [*PSI* ^+^] aggregates leading to loss of [*PSI* ^+^] [29].

Modulation of levels and mutations in Hsp70 chaperones and their co-chaperones have various effects on [*PSI* ^+^] and [*URE3*] [30], [31]. For example, Ssa1/2 in excess cures [*PSI* ^+^] [32] and overexpression of Ssa1 cures [*URE3*] [33]. Depletion of Sis1, an Hsp40 J-protein co-chaperone of Hsp70 specifically cures cells of [*PSI* ^+^], [*PIN* ^+^], [*URE3*] and [*SWI^+^*] and leads to an increase in the size of SDS-resistant Sup35 polymers derived from [*PSI* ^+^] aggregates [34]–[37]. It has been suggested that Ssa1/2 and Sis1 recruit [*PSI* ^+^] aggregates to Hsp104 for fragmentation, and that prion stability and propagation are mediated by the chaperone composition of prion aggregates [38]–[40]. Overexpression of the Hsp70 nucleotide exchange factor Sse1 or the Hsp40 chaperone Ydj1 cures [*URE3*] [41], [42].

When aggregated in the [*PSI* ^+^] state, Sup35's participation in translation termination is greatly reduced. This leads to increased read-through of stop codons, including the *ade1-14* nonsense allele that can be readily monitored by a red/white color assay [16], [43]–[45]. [*PSI* ^+^] prion variants or strains manifest a range of distinct prion conformations that differ in levels of Sup35 aggregation and, consequently, in the frequency of stop-codon read-through. Since weak [*PSI* ^+^] variants cause less read-through of stop codons, are less mitotically stable and contain more of the soluble non-prion form of Sup35 than strong [*PSI* ^+^] variants, the color assay allows their distinction by the degree of red pigment accumulated [44], [46]–[49]. Also, these [*PSI* ^+^] variants differ in the size of their SDS-resistant Sup35 polymers [20].

When full length Sup35, or just its Q/N-rich prion domain, is transiently overproduced in [*psi* ^−^] cells, [*PSI* ^+^] is induced to appear, presumably because the excess protein increases the chance that it will form a prion seed [2], [44], [50], [51]. However, efficient *de novo* induction of [*PSI* ^+^] requires the presence of a heterologous prion, e.g. [*PIN* ^+^] [3], [51], [52]. Heritable variants of the [*PIN* ^+^] prion have been distinguished by their efficiency in inducing [*PSI* ^+^] with the inducing efficiency gradually decreasing from very high to high to medium to low [*PIN* ^+^] [53].

In a screen of a high-copy yeast library for genes that enhance [*PSI* ^+^] induction in the absence of [*PIN* ^+^], an excess of any of 11 Q/N-rich proteins was found to promote *de novo* [*PSI* ^+^] appearance upon the overexpression of the prion domain of Sup35 [3]. Similarly, aggregation-prone polyQ sequences could substitute for [*PIN* ^+^] in the case of [*PSI* ^+^] induction, and [*PIN* ^+^] also facilitated the aggregation of proteins with extended polyQ stretches [54]–[56]. It was proposed that the aggregates formed by these Q/N-rich proteins provide a nidus for the formation of the first [*PSI* ^+^] seeds, which then promote Sup35's rapid aggregation. This cross-seeding model postulates a direct interaction between Q/N-rich domains of a newly forming prion and preexisting heterologous prion or prion-like aggregates [3], [54], [57], [58].

Several studies have indicated that heterologous prions or prion proteins can also inhibit prion propagation. For example, some [*PIN* ^+^] variants impede the inheritance of [*PSI* ^+^] [32], [59] and [*PSI* ^+^] and [*URE3*] slightly destabilize each other [33], [53]. Also, overexpression of the Ure2 prion domain or several other fragments of Ure2 cures [*URE3*] [60], and overexpression of some Rnq1 fragments encompassing the Q/N-rich C-terminal domain but lacking the N-terminus is inhibitory to [*PSI* ^+^] and [*URE3*] propagation in the presence of [*PIN* ^+^] [61]. Finally, overexpression of *rnq1* N-terminal mutants causes enlargement of [*PSI* ^+^] aggregates leading to loss of [*PSI* ^+^] [62]. The molecular basis of these antagonistic interactions is unknown.

Here we report that overexpression of a number of Q/N-rich proteins can impede the propagation of the Q/N-rich prions, [*PSI* ^+^] and [*URE3*]. Our studies reveal a physical interaction between two such heterologous Q/N-rich protein aggregates and Hsp104. This hinders the availability of Hsp104 to shear prion aggregates, thereby inhibiting prion propagation. In contrast another overexpressed Q/N-rich protein does not sequester Hsp104, but rather appears to cure [*PSI* ^+^] by increasing the level of Hsp104.

## Results

### Overexpression of some Q/N rich proteins that eliminate the [*PIN* ^+^] requirement for the induction of [*PSI* ^+^] also destabilize pre-existing prions

In an unsaturated genetic screen for overexpressed proteins that cure cells of [*PSI* ^+^], the most efficient curing was observed in the presence of a plasmid encoding a Q/N-rich portion of the *CYC8* gene. Strikingly, *CYC8* was one of the 11 genes we previously uncovered in a screen for genes that in high copy substitute for the [*PIN* ^+^] requirement for the *de novo* induction of [*PSI* ^+^] [3]. Therefore we asked if overproduction of the other proteins identified in the [*PSI* ^+^] induction screen would also destabilize pre-existing [*PSI* ^+^]. Of the 11 chromosomal DNA fragments, 8 (*STE18*, *YCK1*, *PIN2*, *URE2*, *PIN3*, *NEW1*, *NUP116* and *LSM4*) encode full-length proteins with Q/N-rich domains, and another 3 encode partial genes: the C-terminal Q/N-rich domains of *PIN4* and *CYC8*, and the N-terminal Q/N-rich domain of *SWI1*, respectively, called here *PIN4C*, *CYC8C* and *SWI1N*.

Weak (w) [*PSI* ^+^][*PIN* ^+^] was transformed with the 11 multicopy plasmids with the *URA3* and *leu2-d* markers [63], and encoding the above mentioned Q/N-rich proteins and protein fragments. Transformants grown on leucineless media that amplified the plasmids to a high-copy number were then examined for the presence of [*PSI* ^+^] using the color assay. This assay is based on the accumulation of a red pigment in *ade1* mutants and the requirement of the Sup35 protein for proper termination at stop codons: cells in which much of the Sup35 release factor is sequestered into [*PSI* ^+^] aggregates are unable to efficiently terminate translation at the premature stop codon in *ade1-14*, and some full-length Ade1 is synthesized despite the mutation. Thus, *ade1-14* cells that give rise to white or pink colonies are [*PSI* ^+^], while those that grow into red colonies are [*psi* ^−^].

**Figure 1:**
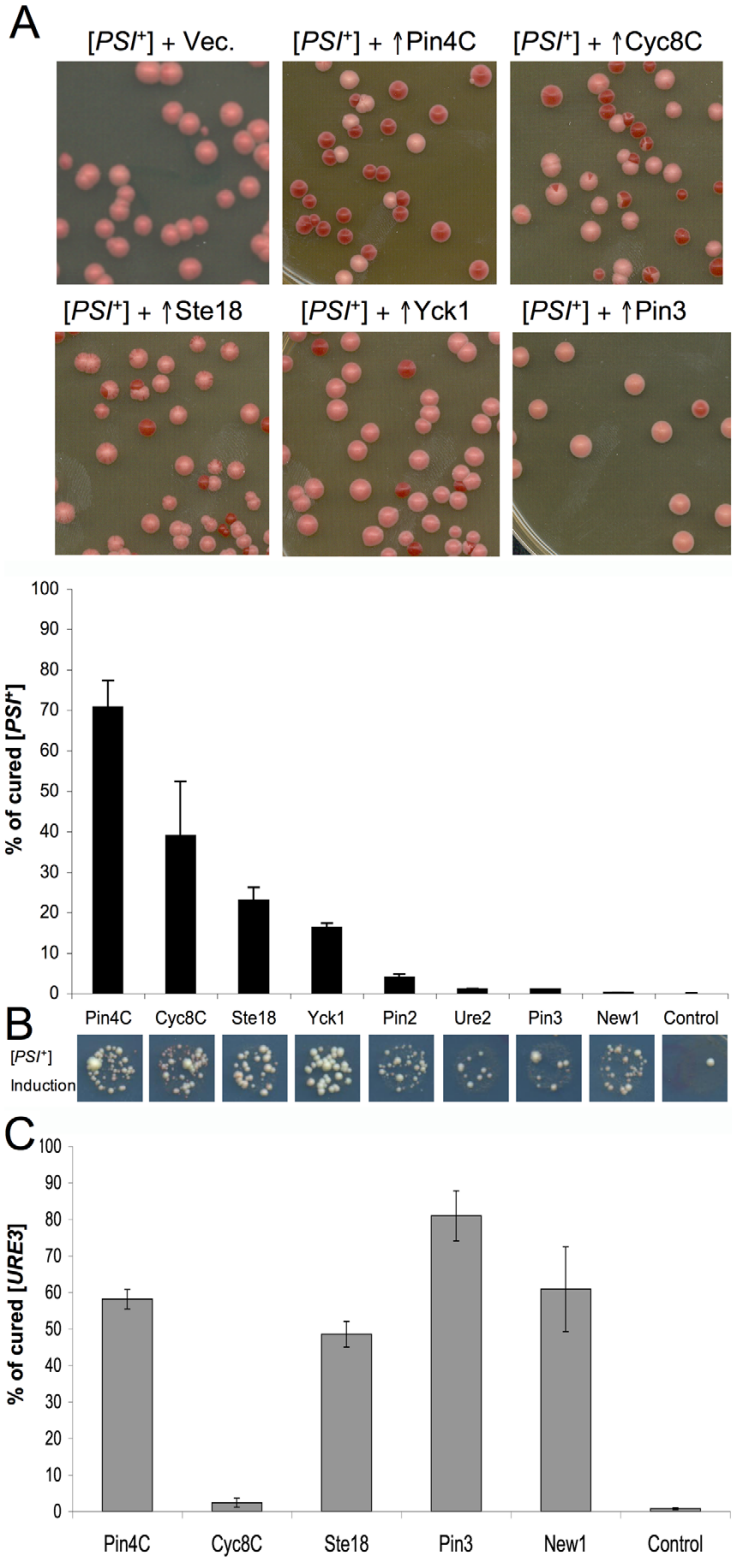
High copy plasmids that enhance induction of [*PSI* ^+^] also cure pre-existing [*PSI* ^+^] and [*URE3*]. (A) Overexpressed Q/N-rich proteins cause loss of [*PSI* ^+^]. Weak (w) [*PSI* ^+^][*PIN* ^+^] (L1758) was transformed with plasmids encoding the indicated Q/N-rich proteins or fragments (*PIN4C* and *CYC8C*), or with the empty vector pHR81. Representative images of transformants plated on YPD following amplification of plasmids on SD-Leu are shown (upper). The efficiency of curing (lower) was determined as the percentage of red colonies indicative of [*psi* ^−^] among ∼1100 colonies. (B) Induction of [*PSI* ^+^] in a *rnq1Δ*::*HIS3* [*psi* ^−^][*pin* ^−^] 74-D694 strain (L3125) carrying the same plasmids as in (A). [*PSI* ^+^] was induced by overexpression of *SUP35NM-GFP* from p*CUP1*-*SUP35NM::GFP-TRP1* in 50 µM Cu^2+^ following library plasmid amplification on SD-Leu. Shown is growth on SD-Ade, which indicates the presence of [*PSI* ^+^]: spots are representative of three repeated experiments. The Ade^+^ colonies were verified to be [*PSI* ^+^] by visualization of Sup35NM-GFP dots. (C) Overexpressed Q/N-rich domains cause [*URE3*] curing. The [*URE3*] derivative of 74D-694 [*PIN* ^+^][*psi* ^−^] expressing the GFP tagged endogenous Sup35 (L3154), was transformed with high copy plasmids encoding the Q/N-rich proteins or protein fragments, or with the empty vector pHR81. The percentage of cured [*ure-o*] cells among ∼1000 colonies was determined using the color assay described in *Materials and Methods*. Error bars show standard error of the mean.

A high proportion of red colonies indicated that amplification of plasmids encoding Pin4C, Cyc8C, Yck1 and Ste18, but not Pin2, Pin3, Ure2 and New1, caused efficient loss of w[*PSI* ^+^] (Figure 1A). Representative red colonies were confirmed to be [*psi* ^−^], because they exhibited diffuse fluorescence after being crossed to a [*psi* ^−^] tester strain carrying Sup35NM-GFP [64], [65]. Furthermore, the resulting [*psi* ^−^] state remained unchanged after elimination of the library plasmids, confirming that [*PSI* ^+^] was indeed lost, not just transiently inhibited. This [*PSI* ^+^] loss is not caused by a growth advantage of [*psi* ^−^] over [*PSI* ^+^] cells when the Q/N-rich domains are overexpressed (Figure S1). Overexpression of Swi1N, Nup116 and Lsm4 caused growth inhibition in w[*PSI* ^+^][*PIN* ^+^] cells, thus impeding analysis of curing of [*PSI* ^+^] by those proteins.

Curiously, the overexpressed plasmids (*PIN4C*, *CYC8C*, *STE18*, *YCK1*) that caused the most efficient curing of w[*PSI* ^+^] also caused efficient induction of [*PSI* ^+^] (Figure 1B). The efficiencies of [*PSI* ^+^] induction were examined in a [*psi* ^−^][*pin* ^−^] *rnq1Δ* strain by assaying read-through of the premature stop codon in the *ade1-14* allele, which was detected as growth on SD-Ade (see *Materials and Methods*).

### [*URE3*] is also destabilized by high copy plasmids encoding Q/N-rich domains

We next examined if the Q/N-rich proteins could cure cells of another Q/N-rich prion, [*URE3*]. A [*URE3*][*PIN* ^+^][*psi* ^−^] derivative of 74-D694 with *SUP35* endogenously tagged with GFP was used. In this background, cells are light red in the absence of [*URE3*], but become dark red when they are [*URE3*] [66].

Overproduced Pin4C and Ste18, which caused efficient curing of w[*PSI* ^+^], caused 58% and 49% loss of [*URE3*] respectively (Figure 1C), indicating that they each have a destabilizing effect on different Q/N-rich prions. However, overproduced Cyc8C, another efficient [*PSI* ^+^] curer, destabilized [*URE3*] only slightly, while Pin3 and New1, which did not have a significant effect on w[*PSI* ^+^] propagation, caused 81% and 61% loss of [*URE3*] respectively (Figure 1C). These distinctions in the ability of different Q/N-rich proteins to cure cells of [*PSI* ^+^] and [*URE3*] imply that they are curing prions via distinct mechanisms.

### Medium and very high [*PIN^+^*], but not high [*PIN^+^*], variants are destabilized by overexpression of Pin4C

Pin4C was chosen for further investigation of how overexpressed Q/N-rich domains cure cells of prions, since it was the most efficient in curing both w[*PSI* ^+^] and [*URE3*]. We thus asked if overexpression of Pin4C could affect propagation of [*PIN^+^*] and found that overexpression of Pin4C caused 30% loss of medium [*PIN^+^*], 8% loss of very high [*PIN^+^*], but had no effect on high [*PIN^+^*] (see *Materials and Methods*). All [*PIN^+^*] strains used in the study of curing of [*PSI* ^+^] and [*URE3*] in this paper were high [*PIN* ^+^].

### [*PSI* ^+^] aggregates increase in size upon overexpression of Pin4C

*SUP35-GFP* strains expressing GFP tagged Sup35 in the original chromosomal location under the control of the *SUP35* promoter were employed to allow for real-time visualization of [*PSI* ^+^] aggregates as the cells were cured of [*PSI* ^+^] by Pin4C overexpression. In [*PSI* ^+^] cells Sup35-GFP accumulates in numerous small cytoplasmic foci. It was previously shown that this *SUP35-GFP* construct is functional, i.e. it can replace the essential Sup35 protein and can stably propagate strong [*PSI* ^+^] [21], [67]. We proceeded with strong [*PSI* ^+^] because w[*PSI* ^+^] was somewhat unstable in the presence of the Sup35-GFP replacement. Thus, all [*PSI* ^+^] strains used in the rest of the paper were strong [*PSI* ^+^].

To tightly control Pin4C overexpression, a plasmid bearing *PIN4C* driven by the inducible *GAL* promoter was introduced into a strong [*PSI* ^+^][*PIN* ^+^] *SUP35-GFP* strain (GF657). Overexpression of *PIN4C* on this *leu2-d* amplified plasmid (pHR81*GAL*-*PIN4C*) caused a 99% loss of strong [*PSI* ^+^] in this [*PIN* ^+^] strain with endogenously tagged *SUP35-GFP*. Likewise, strong [*PSI* ^+^] was also efficiently cured (45% loss) by overexpressing Pin4C (using pHR81H-*PIN4C*) in 74D-694 with untagged Sup35. Consistent with our previous findings that high [*PIN* ^+^] was not cured by overexpression of Pin4C, we found that [*PIN* ^+^] was still maintained in derivatives of 74D-694 that were cured of strong [*PSI* ^+^] as a result of Pin4C overexpression.

**Figure 2:**
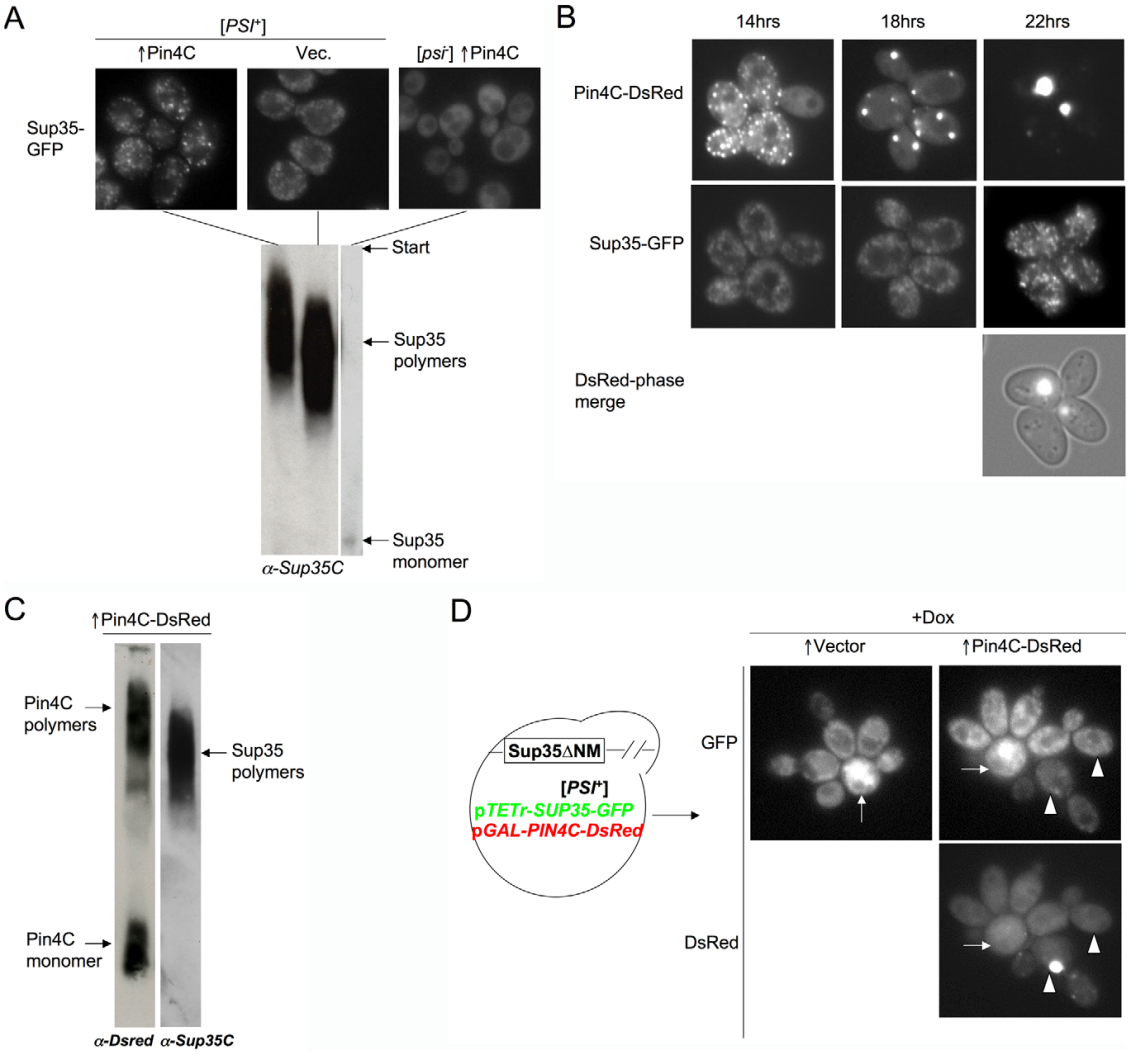
Pin4C overexpression leads to larger [*PSI* ^+^] aggregates. (A) Sup35-GFP aggregates become larger upon overexpression of Pin4C. Upper panels: Representative GFP images of strong [*PSI* ^+^][*PIN* ^+^] *SUP35-GFP* expressing strains (GF657) after an overnight induction of *PIN4C*; empty vector control was incubated in galactose medium for the same amount of time; [*psi* ^−^] culture shown is after 4-days of induction of *PIN4C*. Lower panels: Lysates prepared from cultures shown above were treated with 2% SDS at room temperature and analyzed for the presence of Sup35 by SDD-AGE with anti-Sup35C-GFP. (B) Overexpression of Pin4C-DsRed leads to the formation of single dot-like aggregates concomitant with enlargement of Sup35-GFP aggregates. Representative DsRed and GFP images are of strong [*PSI* ^+^][*PIN* ^+^] *SUP35-GFP* (GF657) cells after induction of pHR81*GAL*-*PIN4C-DsRED* for the times indicated. Cells were grown in liquid plasmid selective galactose media. (C) Pin4C-DsRed aggregates are composed of SDS-resistant polymers. Lysates from cultures where ∼60% of cells contain large single Pin4-DsRed dots were treated with 2% SDS at room temperature and analyzed by SDD-AGE. The blot was probed with anti-DsRed antibody (left lane), stripped and re-probed with anti-Sup35C antibody (right lane). (D) Increased size of visible [*PSI* ^+^] aggregates requires continuous synthesis of the Sup35 protein. Single cells of strong [*PSI* ^+^][*PIN* ^+^] expressing *SUP35ΔNM* at its endogenous locus and harboring extrachromosomal p*TET^r^*-*SUP35-GFP* (L3126) were transformed with pHR81*GAL*-*PIN4C*-*DsRED*, or vector, pHR81*GAL*-*DsRED*. Cells were grown in 2% raffinose +2% galactose +0.025 µg/ml doxcycline for 6 hrs, which induced *PIN4C-DsRED* and allowed the Sup35-GFP level to be close to the normal Sup35 level. Single cells with diffuse DsRed were then micromanipulated and grown for 18 hrs dividing ∼3 times on 2% raffinose +2% galactose +10 µg/ml doxcycline medium where new synthesis of Sup35-GFP is repressed. GFP and DsRed images of a representative part of a growing microcolony are shown. The arrows point to mother cells, and the arrowheads point to two daughter cells with and without a large DsRed dot.

Changes in the state of strong [*PSI^+^*] caused by excess Pin4C were monitored over time. After overnight overexpression of Pin4C, the Sup35-GFP foci became brighter, bigger and more distinct in ∼80% of the cells in comparison with the numerous tiny Sup35-GFP foci formed in control cells lacking the Pin4C plasmid (Figure 2A). Likewise, Pin4C overexpression caused the size distribution of SDS-resistant [*PSI* ^+^] polymers to shift dramatically to larger complexes (Figure 2A). These enlarged Sup35-GFP foci that formed following overnight expression of Pin4C were still capable of propagating [*PSI* ^+^] if expression of Pin4C was turned off. However, when Pin4C was expressed for another 4 days, Sup35-GFP became diffuse and [*PSI* ^+^] was lost (Figure S2).

### Overexpressed Pin4C forms amyloid-like aggregates, which do not colocalize with [*PSI* ^+^] aggregates

As expected of a [*PSI^+^*]-promoting Q/N-rich protein, overexpression of *PIN4C* leads to formation of Pin4C aggregates. When *PIN4C-*DsRED is expressed from a multicopy plasmid under the control of the *GAL* promoter in strong [*PSI* ^+^][*PIN* ^+^] cells, Pin4C-DsRed aggregates first appear as multiple cytosolic puncta after 14 hrs of overexpression. By 22 hours the Pin4C-DsRed usually forms one large focus per cell (Figure 2B). The formation of the large Pin4C-DsRed dot was always accompanied by the appearance of large Sup35-GFP foci within the cell, but they did not colocalize (Figure 2B). Furthermore, although Pin4C aggregates have the SDS-resistant characteristic of amyloid aggregates (Figure 2C), the sizes of Sup35 and Pin4C SDS-resistant polymers were not identical (Figure 2C), suggesting that Pin4C is not a component of SDS-resistant [*PSI* ^+^] polymers.

### *PIN4C*-induced increase of [*PSI* ^+^] aggregate size requires continuous synthesis of Sup35

The large Sup35 aggregates that appeared in the presence of excess Pin4C could have been formed by the simple association of existing [*PSI* ^+^] aggregates, or by enlargement of individual aggregates, e.g. due to reduced shearing of growing amyloid fibers. The first possibility is modeled on our previous finding that overexpressed Sup35 causes pre-existing [*PSI* ^+^] aggregates to coalesce into larger particles [68]. Thus we reasoned that overproduced Pin4C might “glue” existing [*PSI* ^+^] aggregates together through heterologous Q/N-rich domain interactions.

To test this, we examined the appearance of pre-existing [*PSI* ^+^] aggregates when Pin4C was overexpressed. Pre-existing aggregates were made of protein encoded by extrachromosomal *SUP35-GFP* controlled by the repressible *TET^r^* promoter in a strain lacking the endogenous *SUP35* prion domain (*SUP35ΔNM*). After 6 hrs of expression of the *GAL* controlled *PIN4C-DsRED* (i.e. before diffuse Pin4-DsRed formed big foci and before any changes in [*PSI^+^*] aggregates could be noted in previous experiments), single cells were micromanipulated onto solid galactose medium containing doxycycline, where expression of Sup35-GFP was repressed. Later, in cells where large Pin4-DsRed foci appeared, the Sup35-GFP aggregates were still tiny, just as in cells with diffuse Pin4-DsRed examined at the same time (Figure 2D). The faint appearance of the GFP aggregates was due to lack of newly synthesized Sup35-GFP. The absence of large Sup35-GFP foci even in cells with large Pin4C-DsRed aggregates, suggests that the large Sup35-GFP foci seen in the presence of continued Sup35-GFP synthesis are not formed by coalescence of previously formed [*PSI* ^+^] aggregates. Rather, [*PSI* ^+^] aggregates appear to become larger by continuously incorporating newly synthesized Sup35-GFP upon overexpression of Pin4C.

### Pin4C overexpression reduces Sup35-GFP aggregate mobility and transmission to daughter cells

**Figure 3:**
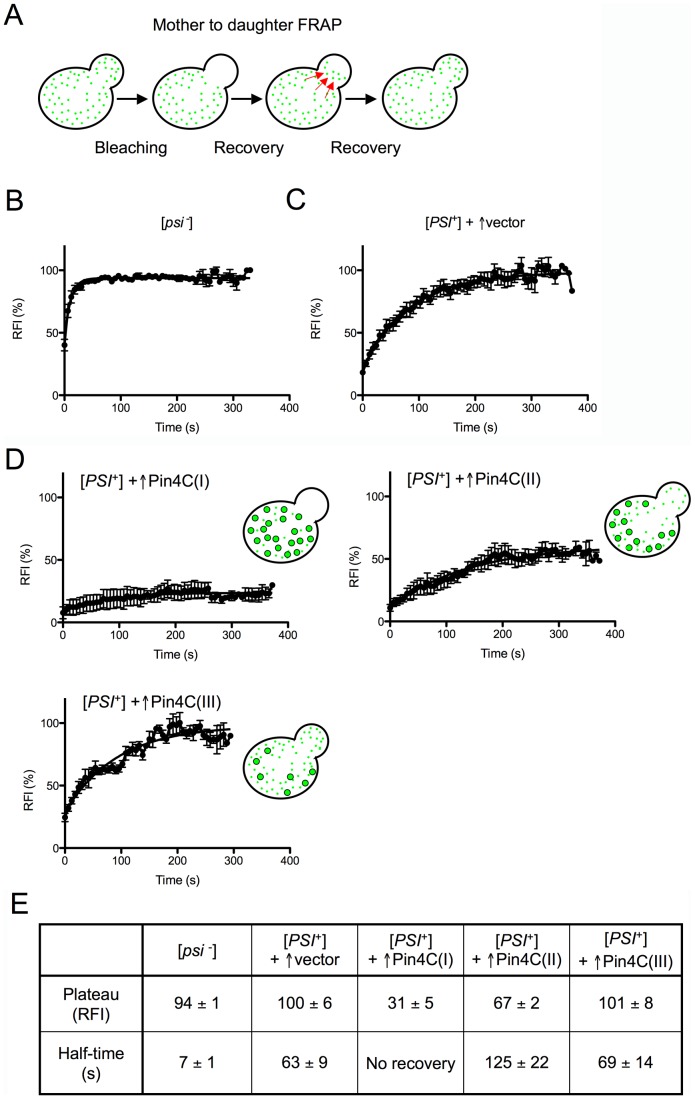
Overexpressed Pin4C reduces the transmission of Sup35-GFP from mother to daughter cells. (A) Diagram of experiment. Fluorescence in daughter cells was photobleached and the time course of fluorescence recovery of the daughter cells was measured as described previously [70]. (B–D) Quantitative FRAP analysis of Sup35-GFP in 10 [*psi* ^−^][*pin* ^−^] (GF658) cells (B), 9 strong [*PSI* ^+^][*PIN* ^+^] (GF657) cells harboring the pHR81GAL vector (C), and 12 strong [*PSI* ^+^][*PIN* ^+^] (GF657) cells harboring pHR81GAL-*PIN4C* and containing enlarged Sup35-GFP foci following overnight Pin4C overexpression (D). The relative fluorescence intensity (RFI) of the bleached daughter cell was determined every 5 s after completion of photobleaching and normalization. Error bars indicate the standard error of the mean. RFI in (D) represents the average of 3 cells (I), 4 cells (II) and 3 cells (III). The analysis of two more cells is not shown. (E) The recovery plateau level and half-time from the curves in B–D are listed.

The dynamics of Sup35-GFP in dividing [*PSI* ^+^] cells upon Pin4C overexpression was probed using fluorescence recovery after photobleaching (FRAP). The rate of transfer of Sup35-GFP from mother to daughter cells was examined by first completely photobleaching daughter cells and then measuring the fluorescence recovery of the daughter (Figure 3A). As shown previously [69], [70], soluble Sup35-GFP in [*psi* ^−^] cells was much more mobile than predominantly aggregated Sup35 in [*PSI* ^+^] cells; the average half-time for recovery in isogenic [*psi* ^−^] versus [*PSI* ^+^] daughters was, respectively, 7 s versus 63 s (Figure 3B, 3C and 3E).

The fluorescence recovery measured following Pin4C overexpression in 12 [*PSI* ^+^] cells containing large Sup35-GFP foci indicated that the population of Pin4C expressing cells is heterogeneous. Three cells exhibited almost no recovery, indicating a major defect in transmission of Sup35-GFP (Figure 3D (I) and 3E). In another 4 cells, Sup35-GFP only recovered to 67% of the intensity observed prior to photobleaching, with a half-time of 126 s that is twice as long as in [*PSI* ^+^] without Pin4C overexpression (Figure 3D (II) and 3E). In one cell 98% recovery was completed with a half-time of 243 s (data not shown). Yet, in 3 cells, fluorescence recovered to 100% with half-times similar to that in [*PSI* ^+^] cells without Pin4C (Figure 3D (III) and 3E). We also observed one cell (data not shown) exhibiting 83% recovery with a half-time of only 17.79 s.

The slow flux of Sup35-GFP in 8 of these mother-daughter pairs indicates that Sup35-GFP often becomes extremely immobile following overexpression of Pin4C, which is consistent with an increase in Sup35 aggregate size and a reduction in the segregation of prion seeds to daughters. However, the presence of cells with normal recovery suggests that at least in some cells, in addition to the large Sup35-GFP foci, there were still small prion seeds available to be transmitted to daughters. Finally, the existence of cells with very fast flow of Sup35-GFP from mother to daughter cells indicates a high level of soluble Sup35 that might be already inefficiently sequestered by the few large Sup35 aggregates still remaining in the mother cell. Differences in the rates of Sup35-GFP transfer among individual mother-daughter pairs suggest that overexpressed Pin4C creates heterogeneity in the properties of prion aggregates during the curing process.

### Microcolonies overexpressing Pin4C show progressive loss of [*PSI* ^+^]

**Figure 4:**
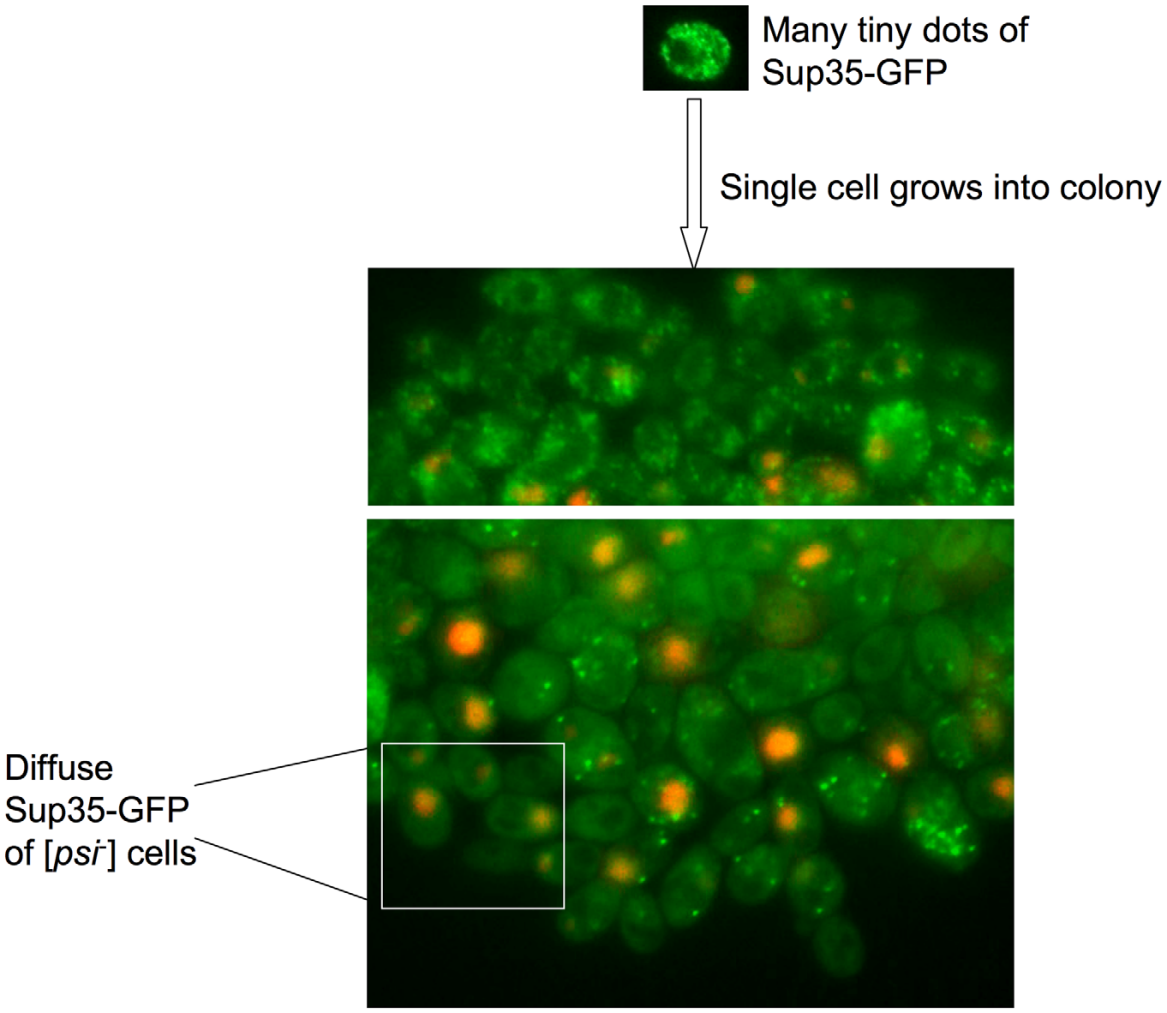
Microcolonies overexpressing Pin4C-DsRed show progressive loss of [*PSI* ^+^]. Single strong [*PSI* ^+^][*PIN* ^+^] *SUP35-GFP* expressing (GF657) cells carrying pHR81*GAL*-*PIN4C-DsRED* were micromanipulated and grown on 2% raffinose +2% galactose to induce Pin4C-DsRed for ∼24 hrs. Portions of a microcolony are shown as the merge of GFP and DsRed images.

To further assess how the appearance of large immobile Sup35 aggregates caused by excess Pin4C correlates with the loss of [*PSI* ^+^], individual cells of the [*PSI* ^+^] Sup35-GFP strain carrying pHR81GAL-*PIN4C-DsRED* were micromanipulated and grown on 2% raffinose + 2% galactose plates where the DsRed tagged Pin4C was expressed. As the cells divided we monitored the changes of Sup35-GFP distribution in the cells within the microcolonies. The outer edges of microcolonies with a single layer of cells were imaged since the central portion of the microcolony included multiple layers of overlapping cells. In the edge of one sector (Figure 4, upper panel), Sup35-GFP remained in the multiple tiny foci seen in [*PSI* ^+^] cells prior to Pin4C induction. But in the edge of another sector (Figure 4, lower panel), Sup35-GFP foci increased in size and were reduced in number progressively in dividing cells, which eventually segregated out [*psi* ^−^] cells (also see Figure S3). Different phenotypes observed in different sectors may be due to differences of *PIN4C* plasmid copy number within individual cells.

### Cell division is required for overexpression of Pin4C to cure [*PSI* ^+^]

**Figure 5:**
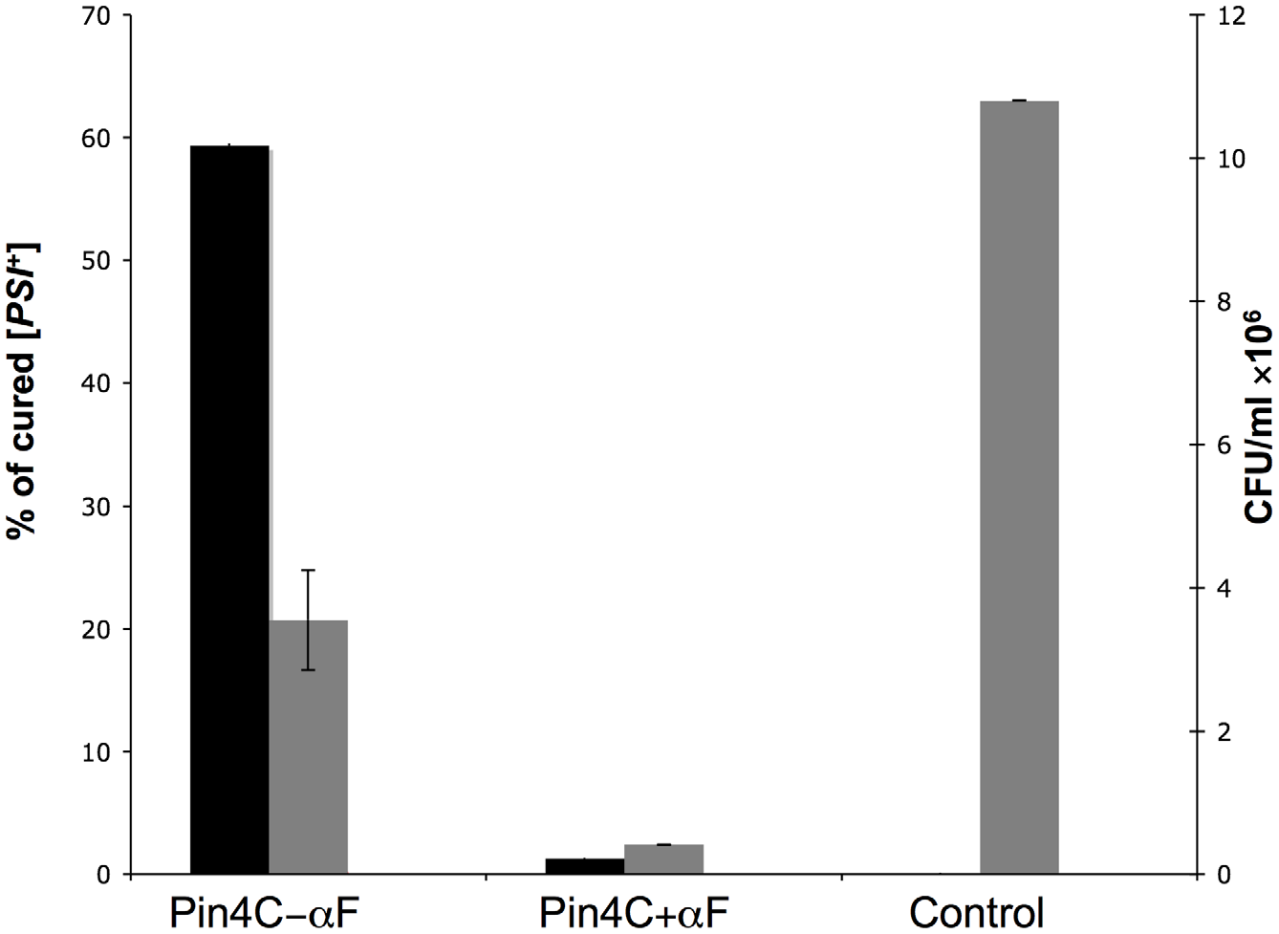
Curing of [*PSI* ^+^] by Pin4C depends on cell division. Three *MAT*a strong [*PSI* ^+^][*PIN* ^+^] *SUP35-GFP* (GF845) transformants harboring pHR81*GAL-PIN4C* were grown in liquid galactose media for 40 hrs, when Sup35-GFP foci became larger in size and fewer in number. Then cells were transferred to fresh galactose medium with or without the addition of 50 µM a-factor for another 16 hrs. Three transformants harboring empty vector pHR81-*GAL* were transferred to galactose medium without the addition of 50 µM a-factor as control. Samples were taken, diluted and plated on YPD where the percentage of red cured [*PSI* ^+^] among ∼850 colonies was scored (black bars). There was no [*PSI* ^+^] loss in the control. The number of CFUs is shown by gray bars. Error bars show standard error of the mean.

Previous studies showed that cell division was essential for the loss of the [*PSI* ^+^] prion in GuHCl-treated cells [71]. To test if cell division was required for the overexpression of Pin4C to cure [*PSI* ^+^], loss of [*PSI* ^+^] was compared in a *MAT* **a** strong [*PSI* ^+^] *SUP35-GFP* strain overexpressing Pin4C in the presence and absence of growth arrest induced by α-factor. Pin4C was overexpressed in liquid galactose for 40 hrs, and 50 µM α-factor was added at this stage, i.e. when Sup35-GFP aggregates were larger and fewer in number, but before the emergence of any diffuse [*psi* ^−^] cells. After overexpressing Pin4C for another 16 hrs, cells were plated on YPD to score for [*PSI* ^+^] loss. The α-factor arrest caused an 88% decrease in colony-forming units, CFUs. Cultures whose growth was arrested vs. not arrested respectively showed, 1% vs. 59% loss of [*PSI* ^+^] (Figure 5). Reduced loss of [*PSI* ^+^] during the α-factor arrest indicates that cell division is required for curing of [*PSI* ^+^] by overexpressed Pin4C.

### Overexpression of Pin4C does not change chaperone levels

Since [*PSI* ^+^] propagation is sensitive to optimal levels of chaperones such as Hsp104, Ssa1/2, Ssb1, Sse1 and Sis1 [72]–[74], it seemed possible that excess Pin4C caused a change in levels of chaperones which then led to loss of [*PSI* ^+^]. However, Pin4C overexpression did not cause a significant alteration in levels of Ssa1/2, Ssb1, Sse1 and Sis1 (Figure S4A).

More thorough analysis revealed that the level of Hsp104 in cells following Pin4C overexpression was reduced to 83% of that in cultures not overproducing Pin4C (Figure S4B and Table S1). Because previous studies showed that a heterozygous disruption of *HSP104* has no effect on [*PSI* ^+^] propagation [75], and that loss of [*PSI* ^+^] is only initiated when the Hsp104 levels drop well below 50% of the normal level [76], it appeared unlikely that the slight decrease in Hsp104 level induced by excess Pin4C would cause [*PSI* ^+^] loss. Indeed, a heterozygous disruption of *HSP104* did not facilitate curing of [*PSI* ^+^] in our strains (Figure S4C).

### Overexpressed Pin4C titrates Hsp104-GFP away from the cytoplasm

The increased size of Sup35 polymers seen upon Pin4C overexpression was similar to that seen upon inhibition of Hsp104 due to a block of prion fragmentation [20]. Thus we considered the possibility that excess Pin4C cures [*PSI* ^+^] by titrating Hsp104 away. Since [*PSI* ^+^] aggregates were found to associate with Hsp104 [38], it seemed possible that Pin4C aggregates also harbor Hsp104.

**Figure 6:**
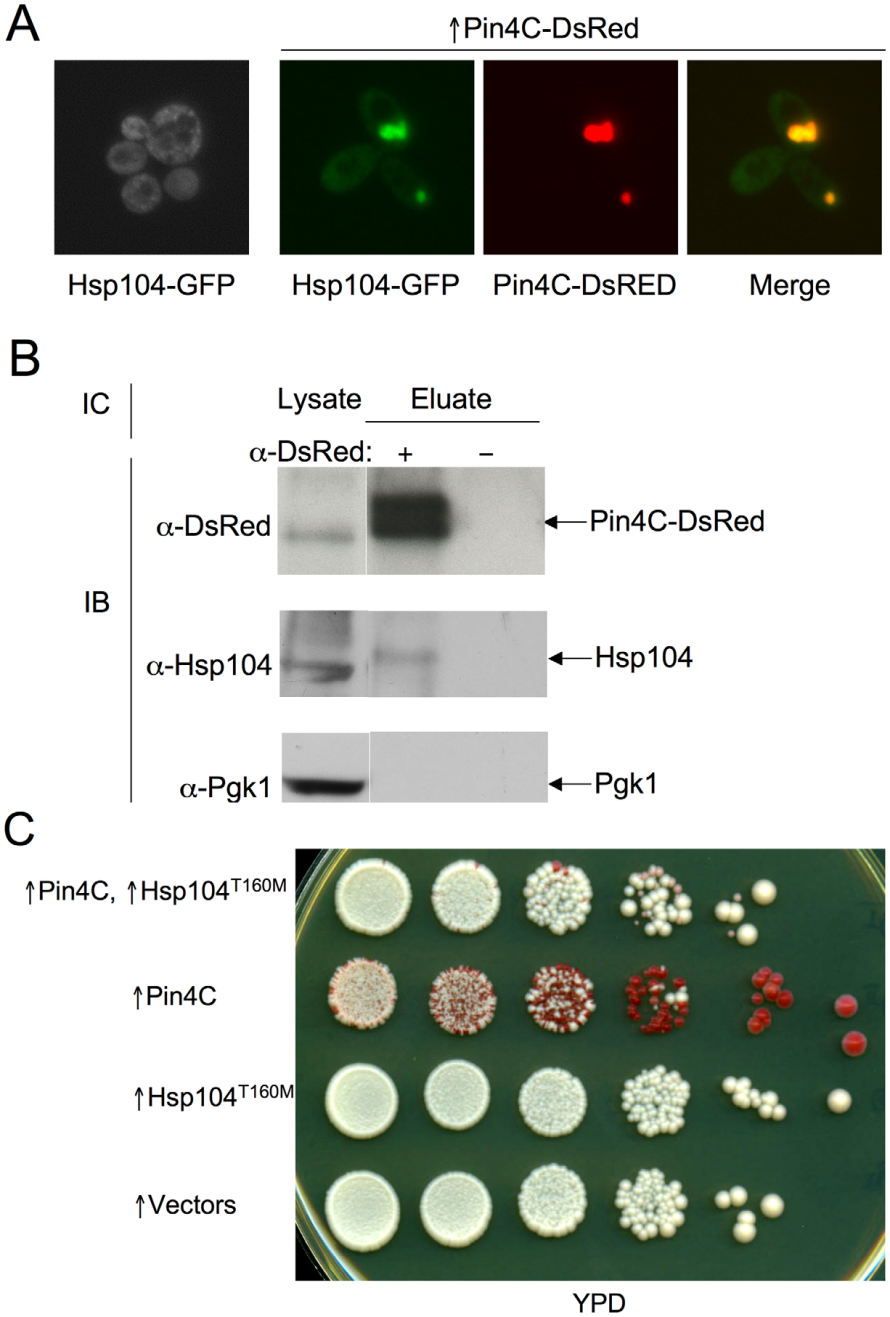
Overexpressed Pin4C sequesters Hsp104 from the cytoplasm. (A) Overexpressed Pin4C sequestered Hsp104-GFP to colocalize with Pin4C-DsRed aggregates. Representative images of cells with endogenous Hsp104 tagged with GFP without or with 16 hrs of induction of pHR81*GAL*-*PIN4C-DsRED* are shown. (B) Overexpressed Pin4C binds to Hsp104. The interaction between Hsp104 and overexpressed Pin4C was assayed in strong [*PSI* ^+^][*PIN* ^+^] (GF657) following overnight overexpression of Pin4C-DsRed. 60 µg of total protein was loaded as the “lysate”. The Pin4C-DsRed complex immunocaptured with anti-DsRed from 500 µg of total protein was loaded as “eluate”. The same membrane was immunoblotted (IB) with anti-DsRed, then with anti-Hsp104, and re-probed with anti-Pgk1 as a control. No co-immunocapture of endogenous Pgk1 with Pin4C was detected, implying that Hsp104 was specifically immunocaptured with the Pin4C complex. The slightly slower migration of Hsp104 in the “eluate” relative to its migration in the “lysate” is probably due to the different buffers used during immunocapture. (C) Overexpression of Hsp104 ^T160M^ suppresses curing of strong [*PSI* ^+^] by Pin4C. Transformants with pHR81*GAL*-*PIN4C* and pRS413*GAL*-*HSP104^T160M^* (↑Pin4C, ↑Hsp104^T160M^); or with pHR81*GAL*-*PIN4C* and empty vector pRS413*GAL* (↑Pin4C); or with pRS413*GAL*-*HSP104^T160M^* and pHR81*GAL* (↑Hsp104^T160M^); or with both empty vectors pHR81*GAL* and pRS413*GAL* (↑vectors) were selected on plasmid selective glucose medium, replica-plated to plasmid selective inducing galactose medium, and then 10-fold serially diluted (10^5^→10^0^ cells from left to right) and spotted onto glucose YPD medium where expression of Pin4C and Hsp104^T160M^ is turned off. There was no growth inhibition in cells overexpressing Pin4C and Hsp104^T160M^ compared to those overexpressing Pin4C alone when spotted on a galactose plate (Figure S5). [*PSI^+^*] loss was scored by the appearance of red [*psi* ^−^] colonies.

**Figure 7:**
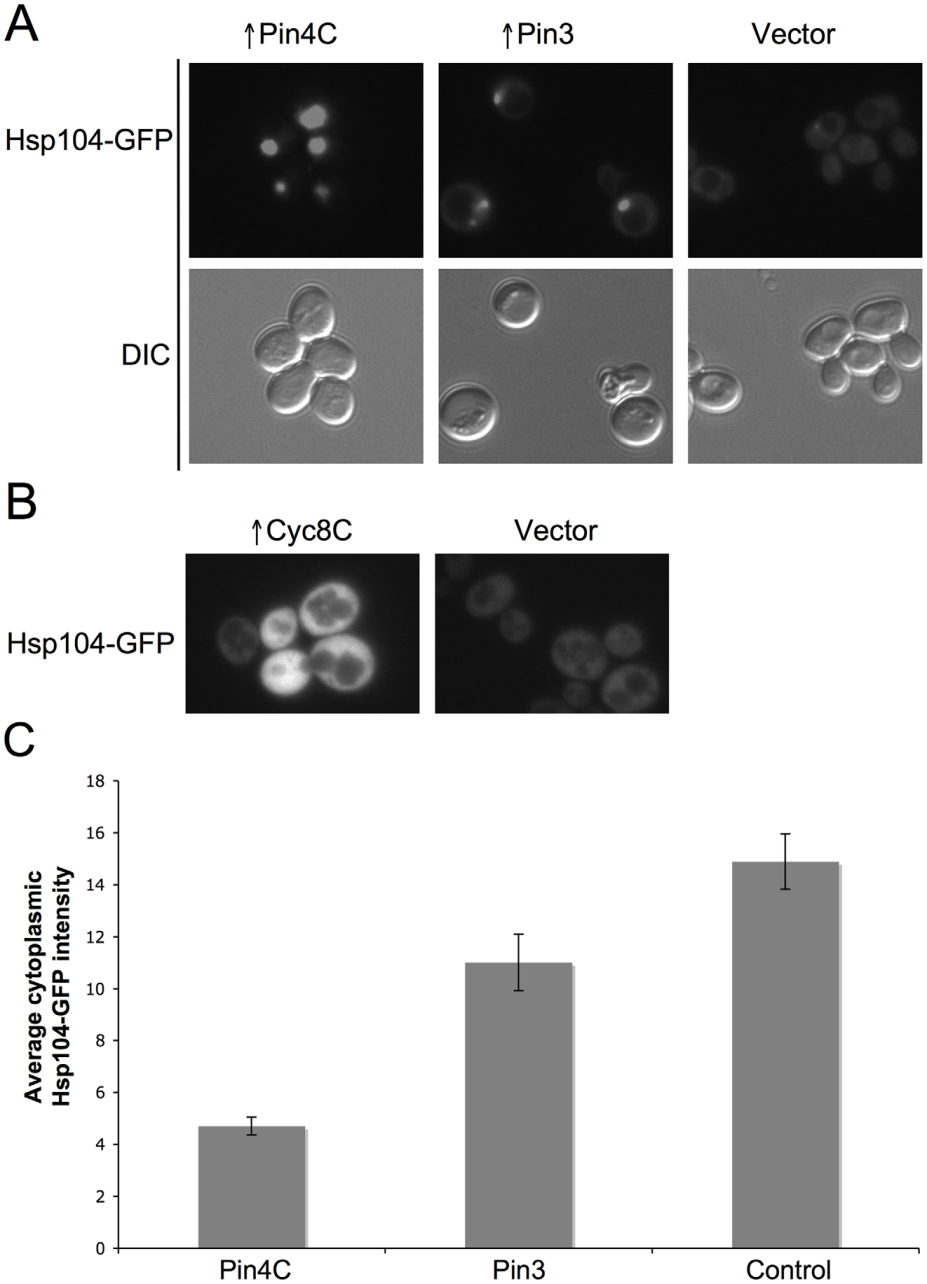
Effects of overexpressed Pin3 and Cyc8C on Hsp104. (A) Overexpressed Pin3 caused endogenous Hsp104-GFP to coalesce into big aggregates. Representative images of Hsp104-GFP cells after induction of pHR81*GAL*-*PIN4C* or pHR81*GAL*-*PIN3* for 16 hrs are shown. All the images shown were taken in the same exposure time. (B) Hsp104-GFP became brighter upon overexpression of Cyc8C. Representative images of Hsp104-GFP cells after 16 hrs induction of Cyc8C from pHR81 based high copy vectors with the insert encoding *CYC8C* or the empty vector on SD-Leu liquid medium. All the images shown were taken in the same exposure time. (C) Overexpressed Pin3 caused the sequestration of Hsp104 less effectively than Pin4C. The bar graphs represent the average of the mean fluorescence signal intensity in cytoplasmic regions devoid of aggregates and excluding the vacuole in 22 Hsp104-GFP cells. Error bars indicate the standard error of the mean. P<0.0001 for comparisons of fluorescence intensity in cells with overexpressed Pin4C or Pin3 and vector control.

To visualize the distribution of Hsp104, we used the Hsp104-GFP strain from the endogenously GFP-tagged yeast library [77]. In unstressed cells, Hsp104-GFP is observed as diffuse GFP or occasionally tiny foci with diffuse background (Figure 6A). However, after 16 hrs of induction of untagged Pin4C, Hsp104-GFP coalesced into one large aggregate per cell. Such large Hsp104-GFP aggregates were never found in control cells without Pin4C overexpression. When Pin4C tagged with DsRed was used, the big Pin4C-DsRed focus colocalized with the coalesced Hsp104-GFP (Figure 6A). Furthermore, Hsp104 was co-immunocaptured with large Pin4C-DsRed aggregates in strong [*PSI* ^+^][*PIN* ^+^] cells (Figure 6B). The sequestration of Hsp104 by overexpressed Pin4C reduced the cytoplasmic level of Hsp104 to about 33% of its normal level (Figure 7) which could have inhibited Hsp104 from shearing [*PSI* ^+^] aggregates and producing new seeds for prion propagation.

### Curing of [*PSI* ^+^] by Pin4C is rescued by elevated levels of Hsp104

If Pin4C sequestration of Hsp104 causes curing of [*PSI* ^+^], elevation of Hsp104 levels should antagonize this. Unlike wild-type Hsp104, overexpression of Hsp104^T160M^ does not cure cells of [*PSI* ^+^]. Also, when expressed at the normal level, Hsp104^T160M^ maintains [*PSI* ^+^] [26]. Therefore we overexpressed the Hsp104^T160M^ mutant allele. Excess Hsp104^T160M^ did not reduce Pin4C aggregation (data not shown), however it reduced the efficiency with which overexpressed Pin4C caused loss of [*PSI* ^+^] (Figure 6C).

### Increased levels of Sis1 prevent aggregation of Pin4C and reduce the ability of Pin4C to cure [*PSI* ^+^]

**Figure 8:**
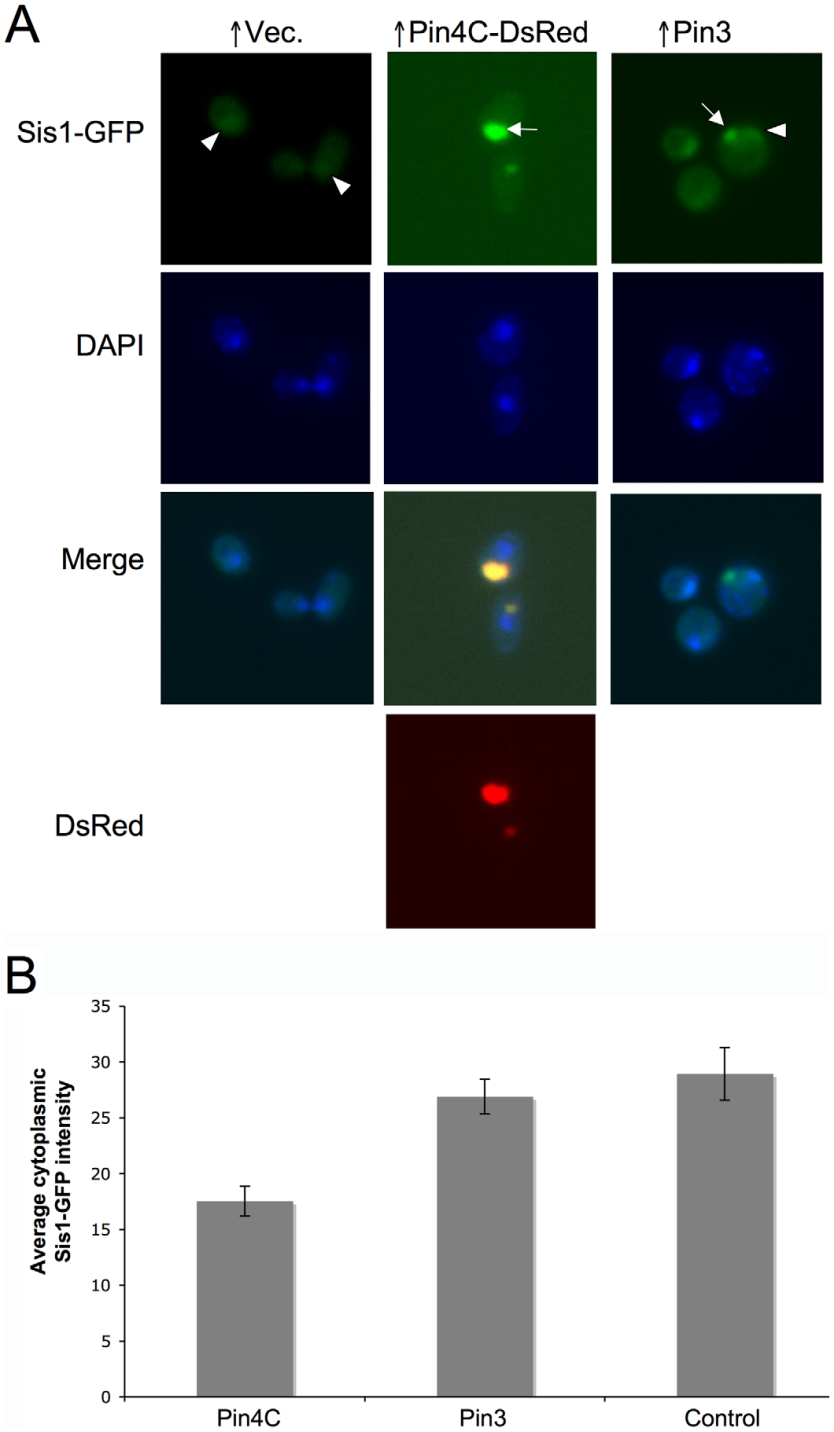
Overexpressed Pin4C or Pin3 caused Sis1 to coalesce. (A) Overexpressed Pin4C or Pin3 causes Sis1-GFP to aggregate. Representative images of endogenous Sis1 tagged with GFP in the absence or presence of 16 hrs of induction of pHR81*GAL*-*PIN4C-DsRED* or pHR81*GAL*-*PIN3* are shown. Fixed cells were permeabilized and stained with DAPI. The arrowhead points to nuclear Sis1-GFP signals, and the arrow points to aggregated Sis1-GFP that is not nuclear. (B) Overexpressed Pin3 did not cause effective sequestration of Sis1 from the cytoplasm. The bar graphs represent the average of the mean fluorescence signal intensity in cytoplasmic regions devoid of aggregates and excluding the vacuole in 20 Sis1-GFP cells. Error bars indicate the standard error of the mean. P<0.0001 for comparisons of fluorescence intensity in cells with overexpressed Pin4C or Pin3 and vector control.

**Figure 9:**
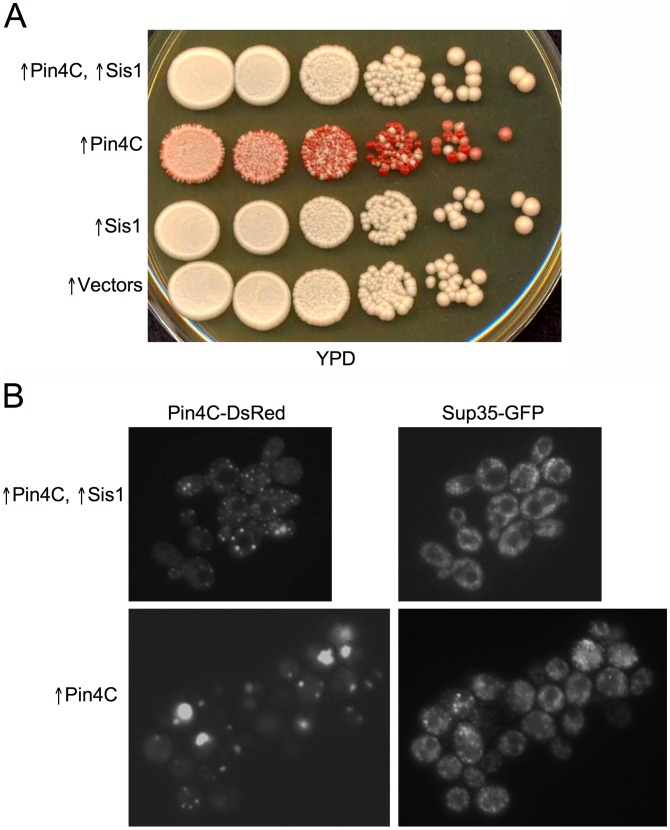
Overexpressed Sis1 reduces elimination of [*PSI* ^+^] by excess Pin4C. (A) Increased levels of Sis1 inhibit [*PSI^+^*] curing by excess Pin4C. Strong [*PSI* ^+^][*PIN* ^+^] (GF657) cells co-transformed with pHR81*GAL*-*PIN4C* and pYES3*GAL*-*SISI* (↑Pin4C, ↑Sis1); or with pHR81*GAL*-*PIN4C* and empty vector pYES3*GAL* (↑Pin4C); or with pYES3*GAL*-*SIS1* and empty vector pHR81*GAL* (↑Sis1); or with two empty vectors pHR81*GAL* and pYES3*GAL* (↑vectors), were examined as described above (see Figure 6C). (B) Overexpressed Sis1 prevents overproduced Pin4C from forming large aggregates. Representative fluorescent images of strong [*PSI* ^+^][*PIN* ^+^] (GF657) cells harboring pHR81*GAL*-*PIN4C-DsRED* and pYES3*GAL*-*SISI* (↑Pin4C, ↑Sis1), or pHR81*GAL*-*PIN4C-DsRED* and empty vector pYES3*GAL* (↑Pin4C) after overnight induction in liquid galactose are shown.

Sis1 is a chaperone involved in cleaving [*PSI* ^+^] aggregates and generating new prion seeds. It was hypothesized to recruit Hsp104 to the sites of prion aggregation [39]. We observed that overexpressed Pin4C also sequestered Sis1. As described previously [78], without Pin4C overexpression most Sis1-GFP was found in the nucleus (Figure 8A). Upon Pin4C-DsRed overexpression, much of the Sis1-GFP colocalized with cytoplasmic Pin4C-DsRed aggregates, and the amount of Sis1-GFP remaining in the nucleus was significantly reduced (Figure 8). Thus we asked if increased levels of Sis1 would affect curing of [*PSI* ^+^] by overexpression of Pin4C. Indeed, we found that the loss of [*PSI* ^+^] by overexpression of Pin4C was significantly reduced by overexpression of Sis1 (Figure 9A). Furthermore, in cells with excess Sis1, overproduced Pin4-DsRed accumulated in several small foci and did not form the huge single focus observed in the absence of excess Sis1. Also, in cells with excess Sis1, Sup35-GFP remained in multiple tiny foci, that did not enlarge upon Pin4C overexpression (Figure 9B). Thus, overproduced Sis1 prevents overexpressed Pin4C from forming big foci and reduces the formation of large Sup35 aggregates, which may cause decreased [*PSI* ^+^] curing by Pin4C.

### Effects of overexpressed Pin3 and Cyc8C on chaperones

To investigate if titrating Hsp104 is a general mechanism by which the heterologous Q/N-rich proteins cure prions, we visualized Hsp104-GFP in cells overexpressing Pin3 or Cyc8C. Like Pin4C, overexpressed Pin3 caused Hsp104-GFP to coalesce into large aggregates and reduced the level of diffuse cytoplasmic Hsp104-GFP fluorescence relative to empty vector controls. However, overexpressed Pin3 also caused a slight increase in the cellular levels of Hsp104, Sse1, Ydj1 and Sis1 (Figure S6A). The combined result of these two effects was that the Hsp104 cytoplasmic level was about 74% of that seen in cells without Pin4C overexpression (Figure 7C). Likewise, although overexpressed Pin3 caused Sis1-GFP to coalesce (Figure 8A), the levels of Sis1 that remained in the cytoplasm were similar to controls (Figure 8B). In contrast to overexpressed Pin4C or Pin3, overexpressed Cyc8C caused an 8-fold increase in the Hsp104 level (Figure S6B), and did not sequester Hsp104 (Figure 7B). Overexpressed Cyc8C also caused a slight increase in the Sis1 level (Figure S6B).

### Overexpressed Gpg1 may titrate chaperones away from the cytoplasm

**Figure 10:**
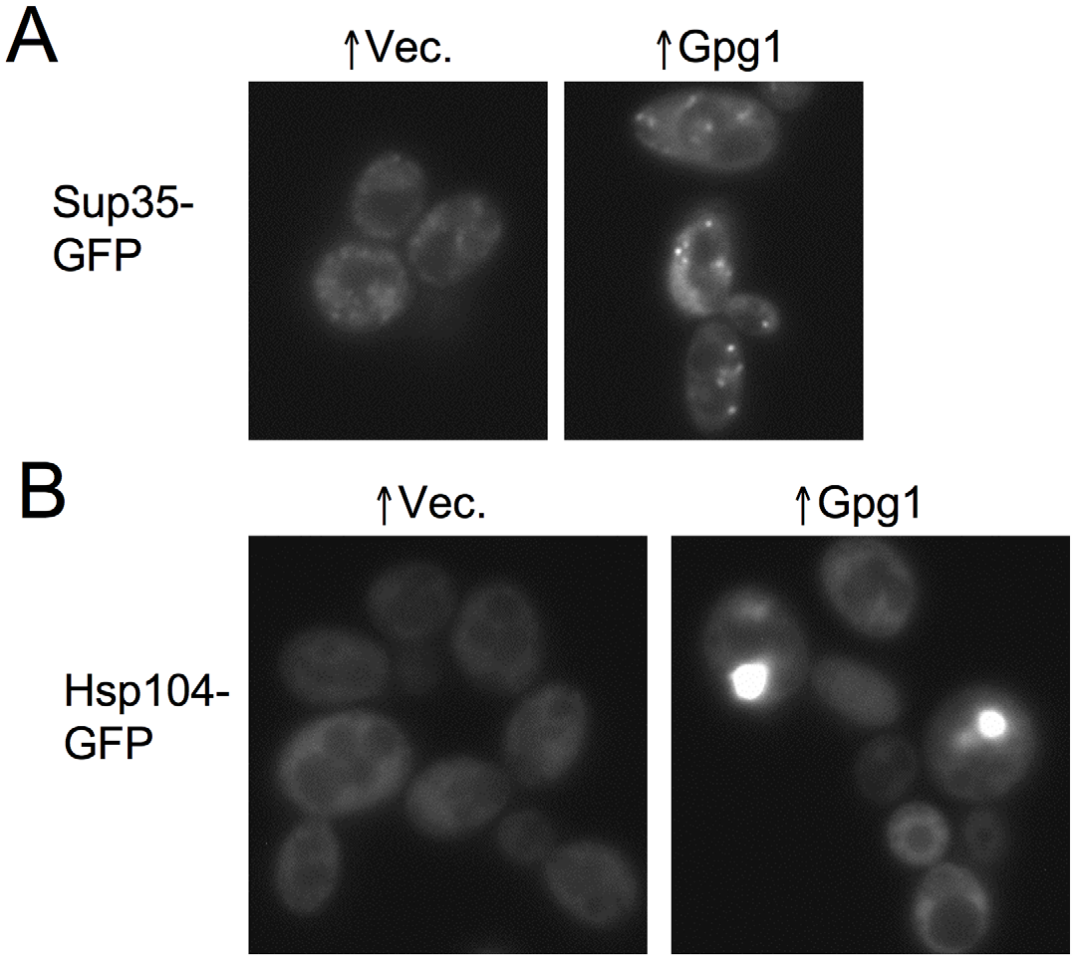
Overexpression of the non-Q/N-rich protein Gpg1 leads to larger [*PSI* ^+^] aggregates and causes Hsp104 to aggregate. (A) Overexpressed Gpg1 leads to larger [*PSI* ^+^] aggregates. Representative GFP images of strong [*PSI* ^+^][*PIN* ^+^] *SUP35-GFP* expressing cells (GF657) transformed with pRS316-*GAL1-GPG1* or empty vector control were of cells incubated overnight in galactose medium for the same amount of time. (B) Overexpressed Gpg1 caused the aggregation of Hsp104-GFP. Representative images of cells with endogenous Hsp104 tagged with GFP with 16 hrs of induction of pRS316-*GAL1-GPG1* or empty vector as control are shown.

We next investigate if non-Q/N-rich aggregates that cure [*PSI* ^+^] also sequester chaperones. Gpg1 is a mimic of a G protein γ subunit. Like overexpressed Pin4C, overexpressed Gpg1 formed aggregates, had reduced curing efficiency when Hsp104 was overexpressed, but did not affect the cellular levels of Hsp104 [79]. Previous work visualizing Gpg1 curing of [*PSI* ^+^] aggregates was complicated by the use of overexpressed Sup35NM-GFP [79]. When we examined the effect of excess Gpg1 on fluorescent aggregates in [*PSI* ^+^] cells with endogenous Sup35 tagged with GFP, we found that the fluorescent dots got larger and fewer in number (Figure 10A), just as seen when Pin4C was overexpressed. Furthermore, like excess Pin4C, excess Gpg1 caused endogenous Hsp104 tagged with GFP to aggregate into foci. However, despite this aggregation we did not detect any reduction in the intensity of cytoplasmic Hsp104-GFP compared to the vector control (Figure 10B).

## Discussion

Surprisingly, several factors that enhance prion induction also cause prion destabilization: [*PIN* ^+^] both facilitates [*PSI* ^+^] appearance and destabilizes [*PSI* ^+^] [51], [59]; overexpression of the Ure2 prion domain both induces *de novo* [*URE3*] appearance and cures [*URE3*] [60]; overexpression of the same chaperones both enhance *de novo* [*URE3*] generation and destabilize existing [*URE3*] [80]. We now report that overexpression of many of the 11 Q/N-rich proteins, which in high copy substitute for the [*PIN* ^+^] requirement for [*PSI* ^+^] induction, also destabilize pre-existing [*PSI* ^+^] or [*URE3*] (Figure 1), indicating that Q/N-rich proteins can also both enhance prion appearance and impair propagation of existing prions.

Our finding that the high copy plasmids encoding 11 Q/N-rich domains can promote the *de novo* induction of [*PSI* ^+^] in an *rnq1*Δ strain, establishes that the induction of [*PSI* ^+^] does not first require the appearance of [*PIN* ^+^]. Rather, this suggests a direct interaction between heterologous Q/N-rich proteins and Sup35, and, as originally hypothesized [3], and as indicated by earlier in vitro studies [54], [58], that the Q/N-rich proteins themselves are likely substituting for [*PIN* ^+^] as the cross-seeds.

Since certain [*PIN* ^+^] variants (e.g. low [*PIN* ^+^], medium [*PIN* ^+^] and very high [*PIN* ^+^] but not high [*PIN* ^+^]) destabilize [*PSI* ^+^], curing of [*PSI* ^+^] by Q/N-rich proteins could result from changing the [*PSI^+^*]-inducing high [*PIN* ^+^] into a destabilizing [*PIN* ^+^]. However, this seems unlikely because the effects of the destabilizing [*PIN* ^+^]s are limited to weak but not strong [*PSI* ^+^] [59], while overexpressed Pin4C also cures strong [*PSI* ^+^]. Furthermore, overexpressed Q/N-rich proteins caused loss of weak [*PSI* ^+^] even without [*PIN* ^+^] (data not shown).

Other previously hypothesized mechanisms of prion curing by Q/N-rich proteins are that the overexpressed Q/N-rich domain of Ure2 inhibits prion fiber growth leading to curing of [*URE3*], either by incorporating into the growing tip of the [*URE3*] seed thereby blocking or “capping” its growth [60], [81], [82], or by sequestering Ure2 preventing it from joining prion fibers [81], [82]. However, this mechanism implies a very efficient interaction between the curing protein and the prion-forming protein, which is likely only in the case of very high homology.

Our studies indicate that overexpression of Pin4C allows [*PSI* ^+^] aggregates to continuously incorporate soluble Sup35, but prevents proper fragmentation (Figure 2, Figure 3, Figure 4, Figure 5). Thus, overproduced Pin4C causes [*PSI* ^+^] loss via a defect in breakage of growing [*PSI* ^+^] fibers and transmission of prion seeds. This is quite different from either the capping or sequestration of its own protein models. Since prion fragmentation is crucially dependent on chaperones, particularly on Hsp104, overexpressed Pin4C could affect the function of Hsp104 on [*PSI* ^+^]. One possibility is that Pin4C coats [*PSI* ^+^] aggregates through the interaction of their Q/N-rich domains and thus shields Sup35 polymers from the shearing activity of chaperones. However, we did not detect co-localization of Sup35-GFP and Pin4C-DsRed foci (Figure 2B).

Although there is no significant alteration in total levels of chaperones (Figure S4), excess Pin4C sequesters Hsp104 and Sis1 (Figure 6, Figure 7, Figure 8), reducing their availability to shear [*PSI* ^+^] aggregates. Although depletion of Sis1 only causes delayed and gradual loss of [*PSI* ^+^] [35], the reduced availability of Sis1 may enhance the effect of Hsp104 sequestration. Furthermore, although excess Sis1 prevented curing of [*PSI* ^+^] by Pin4C overexpression, excess Sis1 also decreases Pin4C aggregation (Figure 9).

We also observed that different Q/N-rich proteins had distinct effects toward the yeast Q/N-rich prions, [*PSI* ^+^] and [*URE3*] (Figure 1). For example, our data is consistent with the recent study showing that overproduction of New1 does not cure [*PSI* ^+^] [83], but we further uncover that overexpressed New1 does cause efficient curing of [*URE3*]. Likewise, Pin3 cures [*URE3*] but not [*PSI* ^+^]. One explanation could be that these proteins sequester Hsp104 less efficiently than the proteins that cure both prions such as Pin4C. Indeed, we found that overexpressed Pin3 reduced the cytoplasmic level of Hsp104 less effectively than Pin4C (Figure 7 and Figure S6A). Since more than 50% of the cellular Hsp104 remained diffuse when Pin3 was overexpressed, curing of [*PSI* ^+^] would not be expected [76]. However, since there are fewer prion seeds in [*URE3*] than weak [*PSI* ^+^] cells [35], this slight reduction in soluble Hsp104 might be sufficient to cure [*URE3*]. Indeed, previous studies showed that different prions and different prion variants have different susceptibilities towards chaperone activities [37], [69], [84]. Although overexpressed Pin3 sequestered Sis1 (Figure 8A), there was no significant difference in the fluorescence intensity of diffuse Sis1-GFP in the cytoplasm with Pin3 overexpression compared to the control without Pin3 overexpression (Figure 8B), suggesting that sequestration of Sis1 by excess Pin3 does not significantly contribute to curing of [*URE3*]. There was also a slight increase in the total cellular levels of Sse1 and Ydj1, which could contribute to the Pin3 curing.

Surprisingly, overexpression of Cyc8C cures [*PSI* ^+^] but not [*URE3*] (Figure 1). This cannot be explained by sequestration of Hsp104. Indeed, overexpression of Cyc8C did not cause aggregation of Hsp104 (Figure 7B), but rather increased the Hsp104 level 8-fold while having no effect on Ssa1 (Figure S6B). Since overexpression of Hsp104 cures [*PSI* ^+^] but not [*URE3*] [16], [42], this provides a plausible mechanism. We also observed a slight increase in the Sis1 level by overexpressed Cyc8C (Figure S6B). It was previously shown that overproduced Sis1 enhances curing of [*PSI* ^+^] by overexpressed Hsp104 [80], therefore overexpressed Cyc8C may cure [*PSI* ^+^] through additive effects of the increased level of Hsp104 and Sis1.

Our findings may provide an explanation for previous observations that overexpressed proteins lead to curing of prions. Overexpression of the Rnq1Δ100 protein (the Q/N-rich C-terminal domain) eliminates [*PSI* ^+^] and [*URE3*] in the presence of [*PIN* ^+^] [61]. Also mutations in the non-Q/N-rich domains of *RNQ1* cause an increase in the size of Sup35 aggregates leading to curing [62]. Both of these phenomena could be because the Rnq1 fragment or mutants form [*PIN* ^+^]-dependent aggregates that sequester Hsp104 and/or other chaperones and reduce their availability to aid [*PSI* ^+^] propagation.

The chaperone titration curing mechanism may also be applicable to non-Q/N-rich aggregates. Indeed, a similar mechanism could explain prion curing caused by overexpression of the non-Q/N-rich protein Gpg1 (Figure 10). Although Gpg1 sequestered Hsp104, we could not detect a reduction in cytoplasmic Hsp104. Nonetheless, other chaperones might also be sequestered by Gpg1 aggregates leading to reduced prion shearing and prion loss.

A recent study also indicates that the cellular localization of chaperones can have a direct impact prion propagation. Indeed, this appears to explain the long-standing mystery of why overexpression of Hsp104 cures cells of [*PSI* ^+^] but not of other prions [16], [36], [42], [51]. Hsp104 overexpression was shown to inhibit shearing of [*PSI* ^+^] aggregates because the excess of Hsp104 displaced the Hsp70 chaperone Ssa1 from the [*PSI* ^+^] aggregate. Hsp104 does not bind to other prions in the absence of Ssa1, consistent with the absence of curing [29].

Other studies also indicate that titration of cellular proteins by amyloid aggregates can have a profound effect on the cell. Indeed, sequestration of essential proteins by amyloid aggregates was previously shown to cause prion toxicity. The large Sup35 aggregate that forms when Sup35 is overexpressed in [*PSI* ^+^] cells sequesters the essential Sup35 binding partner Sup45, resulting in death [68]. Likewise, the large Rnq1 aggregates that form when Rnq1 is overexpressed in [*PIN* ^+^] cells sequester the core spindle pole body component Spc42 causing toxicity [85]. Also, polyglutamine (polyQ) aggregates sequester essential endocytic components such as Sla2 [86] and endoplasmic reticulum associated degradation proteins in [*PIN* ^+^] dependent toxicity [87], and sequester Sup35 in [*PSI* ^+^]-dependent polyQ toxicity [88].

Our results establish sequestration of specific chaperones by overexpressed proteins as a general mechanism to alter the cellular localization of chaperones and therefore inhibit prion propagation. Similar mechanisms could influence phenotypic variation by regulating the balance of chaperones needed for prion propagation, in response to environmental stimuli. Since biochemical pathways controlling prion formation and/or maintenance appear to be conserved from yeast to mammals, titration of chaperones via heterologous Q/N-rich aggregates might provide a new approach to prion and amyloid disease intervention.

## Materials and Methods

### Strains, media, and plasmids

All strains used are described in Table 1. GF657 and GF658, respectively, are strong [*PSI* ^+^][*PIN* ^+^] and [*psi* ^−^][*pin* ^−^] versions of 74-D694 with endogenous *SUP35* replaced with *SUP35-GFP* (SY80 and SY84 from T. R. Serio) [21]. Unless otherwise stated, all strains used in the study are high [*PIN* ^+^] [53]. L3079 was constructed by disrupting chromosomal *RNQ1* of GF657 with *Δrnq1::HIS3*. L3126 was constructed by transforming GF657 with exogenous p*TET^r^*-*SUP35-GFP* to maintain [*PSI* ^+^] and then replacing chromosomal *SUP35-GFP* at its genomic locus with *SUP35ΔNM* by integration and excision using MluI digested p*EMBL-Δ3ATG* [89]. This construct was verified by detection of *SUP35ΔNM* (deletion of amino acid residues 1–253) on a western blot. A [*psi* ^−^][*pin* ^−^] version of strain 64-D697 transformed with p*CUP1-SUP35NM::GFP-TRP1* was crossed to strains to confirm their [*PSI* ^+^] state which was indicated by the appearance of fluorescent foci. GF657 was cured of [*PSI* ^+^] by overexpression of Pin4C to generate L3107, and then grown on ethidium bromide [90] to generate a [*rho* ^−^] petite version L3116. GF827 was grown on 5 mM guanidine hydrochloride to cure [*PSI* ^+^] [91] and [*PIN* ^+^] [52] to generate L3152. Cytoduction from donor L3152 into recipient L3116 gave rise to the 74D-694 [*URE3*][*PIN* ^+^][*psi* ^−^] derivative, L3154. The [*rho* ^+^] cytoductants were confirmed by their inability to grow on medium lacking histidine. GF708 transformed with p*URE2-URE2N::GFP-HIS3* was crossed to strains to score for their [*URE3*] state which was indicated by the appearance of fluorescent foci.

**Table 1:** Yeast strains.

Strain	Genotype	References
64-D697	*MATa ade1-14 ura3-52 leu2-3,112 trp1-289 lys9-A21* [*psi^−^*][*pin^−^*]	[51]
74D-694	*MATa ade1-14 leu2-3,112 his3-*Δ*200 trp1-289 ura3-52*	[50]
L1749	*MATa ade1-14 leu2-3,112 his3-*Δ*200 trp1-289 ura3-52* high [*PIN* ^+^][*psi* ^−^]	[59]
L1758	*MATa ade1-14 leu2-3,112 his3-*Δ*200 trp1-289 ura3-52* weak [*PSI* ^+^] high [*PIN* ^+^]	[50]
L1762	*MATa ade1-14 leu2-3,112 his3-*Δ*200 trp1-289 ura3-52* strong [*PSI* ^+^] high [*PIN* ^+^]	[51]
L1945	*MATa ade1-14 leu2-3,112 his3-*Δ*200 trp1-289 ura3-52* medium [*PIN* ^+^][*psi* ^−^]	[59]
L1953	*MATa ade1-14 leu2-3,112 his3-*Δ*200 trp1-289 ura3-52* very high [*PIN* ^+^][*psi* ^−^]	[59]
L3079	*MATα ade1-14 leu2-3,112 his3-*Δ*200 trp1-289 ura3-52 rnq1Δ :: HIS3 SUP35-GFP* strong [*PSI* ^+^]	This study
L3107	*MATα ade1-14 leu2-3,112 his3-*Δ*200 trp1-289 ura3-52 SUP35-GFP* high [*PIN* ^+^][*psi* ^−^]	This study
L3116	*MATα ade1-14 leu2-3,112 his3-*Δ*200 trp1-289 ura3-52 SUP35-GFP* high [*PIN* ^+^] [*psi* ^−^][*rho* ^−^]	This study
L3125	*MATa ade1-14 leu2-3,112 his3-*Δ*200 trp1-289 ura3-52 rnq1Δ :: HIS3* [*psi^−^*][*pin^−^*]	This study
L3126	*MATα ade1-14 leu2-3,112 his3-*Δ*200 trp1-289 ura3-52 SUP35ΔNM pTETrSUP35-GFP* strong [*PSI* ^+^] high [*PIN* ^+^]	This stud
L3152	*MATa, ura3, leu2, trp1, pDAL5::ADE2 pDAL5::CAN1 kar1* [*URE3*][*psi^−^*][*pin* ^−^]	This study
L3154	*MATα ade1-14 leu2-3,112 his3-*Δ*200 trp1-289 ura3-52 SUP35-GFP* high [*PIN* ^+^] [*URE3*] [*psi* ^−^]	This study
GF657	*MATα ade1-14 leu2-3,112 his3-*Δ*200 trp1-289 ura3-52 SUP35-GFP* strong [*PSI* ^+^] high [*PIN* ^+^]	SY80 [21]
GF658	*MATα ade1-14 leu2-3,112 his3-*Δ*200 trp1-289 ura3-52 SUP35-GFP* [*psi* ^−^][*pin* ^−^]	SY84 [21]
GF708	*MATa ade1-14 his3-Δ200 ura3-52 leu2-3,112 trp1Δ lys2* [*psi^−^*][*pin^−^*]	Cured version of GT81-1C [97]
GF827	*MATa, ura3, leu2, trp1, pDAL5::ADE2 pDAL5::CAN1 kar1* [*URE3*][*PSI^+^*][*PIN* ^+^]	BY241 [98]
GF844	*MATα ade1-14 leu2-3,112 his3-*Δ*200 trp1-289 ura3-52 HSP104::LEU2 SUP35-GFP* [*psi* ^−^]	SY97 [75]
GF845	*MATa ade1-14 leu2-3,112 his3-*Δ*200 trp1-289 ura3-52 SUP35-GFP* strong [*PSI^+^*]	SY81 [21]
GF886	*MATa his3-Δ1 leu2Δ0 met15*Δ*0 ura3Δ0 HSP104-GFP:: HIS3* [*PIN^+^*]	[77]
GF894	*MATa his3-Δ1 leu2Δ0 met15*Δ*0 ura3Δ0 SIS1-GFP:: HIS3* [*PIN^+^*]	[77]

Standard yeast media were used [92]. For overexpression of library high copy plasmids [93] transformants were selected on plasmid selective synthetic media with dextrose lacking uracil (SD-Ura) and then spread on synthetic media lacking both uracil and leucine (SD-Ura-Leu) to amplify the copy number of *leu2-d* bearing plasmids about 100 fold [63]. For Pin4C overexpression from pHR81*GAL*-*PIN4C* in liquid medium, cultures were grown in 2% raffinose synthetic media lacking uracil for ∼8 hrs and then transferred to 2% raffinose + 2% galactose media lacking uracil and leucine (SGal-Ura-Leu) for ∼16 hrs. Transformants carrying double plasmids were selected on SD-Ura-Trp and replicated to SGal-Ura-Trp-Leu to induce overexpression of the *GAL* controlled genes on both plasmids.

### Plasmids

p*CUP1-SUP35NM::GFP-TRP1* carries fusions of amino acids 1–254 of Sup35 and GFP under the *CUP1* promoter [3]. p*URE2-URE2N::GFP-HIS3* (a kind gift from R. B. Wickner) carries fusions of amino acids 1–65 of Ure2 and GFP under the *URE2* promoter. The high copy genomic library used in our earlier study was constructed in the pHR81 (2 µ *URA3 leu2-d*) vector [3], [93]. The *PIN4C Cla*I-*Xho*II fragment isolated from the *PIN4* library clone #277 [3] was Klenow-filled and cloned into pHR81H (2 µ *HIS3 leu2-d*, with the *URA3* marker of pHR81 exchanged for the *HIS3* marker) vector at the blunt-ended *BamH*I site to generate pHR81H-*PIN4C* (2 µ *HIS3 leu2-d*). The *GAL* promoter isolated from pRS316-*GAL1* (a kind gift from A. Bretscher) on the *Xho*I fragment was filled in and cloned into the unique pHR81 *BamH*I site to create pHR81*GAL*. The *PIN4C* fragment isolated from the *PIN4* library clone #277 [3] as an *Xho*II fragment was filled in and cloned into pHR81*GAL* at the *BamHI* site to generate pHR81*GAL*-*PIN4C*. *DsRED* as a *Nde*I-*Not*I fragment digested from pDsRed-Monomer-N1 vector (Clontech) was filled in and cloned into pHR81*GAL* at the *BamH*I site to produce pHR81*GAL-DsRED*. The *PIN4C* fragment was PCR amplified using primers with *BamH*I linkers and subcloned into pHR81*GAL*-*DsRED* at the *BamH*I site to produce pHR81*GAL*-*PIN4C-DsRED*. The PCR primers were P1 (5′-ggctcgagtggatcggcgggaggaaattgaaag-3′) and P2 (5′agtggatcctcgagtggtacctctagaagtatataataccatagattc-3′). The *SUP35-GFP* fragment isolated as a *Sac*I-*BamH*I fragment from p1744 (SB238, kindly provided by T. R. Serio) was subcloned into p*TETr* vector p1331 (a kind gift of pCM184 from E. Herrero) to create p*TETr*-*SUP35-GFP*. Plasmid p*GAL-HSP104^T160M^* is a kind gift from D. C. Masison [26]. Plasmid *pYES3GAL-SIS1* is a kind gift from S. L. Lindquist [94]. *GPG1* isolated from p*GPD*-*GPG1* (a kind gift from Y. Nakamura and H. Kurahashi) [79] on the *BamH*I-*Xho*I fragment was filled in and cloned into the unique pRS316-*GAL1 BamH*I site to create pRS316-*GAL1-GPG1*.

### Scoring for loss of prions

A weak [*PSI* ^+^][*PIN^+^*] variant of 74-D694 (L1758) was transformed with the pHR81 based high copy vectors with inserts encoding any of 9 Q/N-rich domains: *PIN4C* clone #277, *CYC8C* clone #151, *STE18* clone #299, *YCK1* clone #103, *PIN2* clone #222, *URE2* clone #155, *PIN3* clone #80, *NEW1* clone #39 and *LSM4* clone #288, whose overexpression substitutes for [*PIN* ^+^] in [*PSI* ^+^] induction [3]. For each clone, three transformants selected on SD-Ura were spread on SD-Ura-Leu where the library plasmids were amplified to high copy number because of their poorly expressed *leu2-d* allele [63]. Three equal size colonies were resuspended in water and spread on YPD where the percentage of red [*psi^−^*] colonies was scored. A similar method was used to score for loss of strong [*PSI* ^+^] in the high [*PIN^+^*] variant of 74-D694 (L1762) by overexpression of pHR81H-*PIN4C* (2 µ *HIS3 leu2-d*) among ∼1500 colonies.

In order to confirm that red colonies were [*psi* ^−^], randomly chosen red colonies for each clone that lost the plasmid were crossed to [*psi* ^−^] 64-D697 harboring p*CUP1*-*SUP35NM::GFP-TRP1*. Expression of the Sup35NM-GFP fusion in resulting diploids induced on SD-Ura-Trp containing 50 µM Cu^2+^, decorates prion aggregates as GFP foci in [*PSI* ^+^] while remaining diffuse in [*psi* ^−^] due to the absence of aggregates. To confirm the [*URE3*] prion state, randomly chosen L3154 dark and light red colonies for each clone were crossed to GF708 harboring p*URE2-URE2N::GFP-HIS3*. The resulting diploids made from dark red colonies produced Ure2-GFP foci confirming that they were [*URE3*], and diploids made from light red colonies gave rise to diffuse fluorescence, confirming that they were [*ure-o*].

Strong [*PSI* ^+^][*PIN^+^*] *SUP35-GFP* strain (GF657) was transformed with the pHR81*GAL* empty vector or pHR81*GAL*-*PIN4C*. Three transformants selected on SD-Ura were spread on SGal-Ura-Leu to overexpress Pin4C. Three equal size colonies were resuspended in water and spread on YPD where the percentage of red [*psi^−^*] colonies was scored. The efficiency of curing was determined as the percentage of red colonies indicative of [*psi* ^−^] among ∼2500 colonies. Red colonies were confirmed to be [*psi* ^−^] by their diffuse Sup35-GFP.

Medium [*PIN^+^*][*psi* ^−^] (L1945), high [*PIN^+^*][*psi* ^−^] (L1749) and very high [*PIN^+^*][*psi* ^−^] (L1953) strains were transformed with pHR81 empty vector or pHR81-*PIN4C*. Two transformants selected on SD-Ura were spread on SD-Ura-Leu to overexpress Pin4C. Three equal size colonies of each transformant were resuspended in water and spread on YPD. Ten colonies from each clone were crossed to [*pin* ^−^] 64-D697 harboring p*CUP1*-*RNQ1::GFP-TRP1* and diffused Rnq1-GFP was scored as [*pin^−^*].

### Assays for induction of [*PSI* ^+^]

An *rnq1Δ::HIS3* [*psi* ^−^][*pin* ^−^] 74-D694 strain bearing any of the 9 genomic library plasmids tested for curing of [*PSI* ^+^] was transformed with p*CUP1*-*SUP35NM::GFP-TRP1*. Transformants selected on SD-Ura-Trp medium were diluted, and about 4×10^4^ cells were spotted on SD-Ura-Leu-Trp medium to amplify the *leu2-d* library plasmids followed by replica-plating on SD-Ura-Leu-Trp containing 50 µM Cu^2+^ to induce [*PSI* ^+^]. Then cells were replica-plated on SD-Ade and scored for [*PSI* ^+^]. The resulting Ade^+^ colonies were verified to be [*PSI* ^+^] by the formation of GFP dots following expression of p*CUP1*-*SUP35NM::GFP-TRP1*.

### Fluorescence microscopy

Yeast expressing Sup35-GFP and Pin4C-DsRed were imaged with a Zeiss Axioskop 2 microscope. For time lapse experiments, single cells were micromanipulated onto a 2% agar patch, then covered with a coverslip and placed on 2% raffinose + 2% galactose plates to allow cell growth. The patch with the coverslip in place was then transferred to a glass slide to image the microcolony and was returned to the plate for further growth. For colocalization experiments, yeast expressing Hsp104-GFP were grown overnight in 2% glucose and then induced in 2% raffinose + 2% galactose liquid medium to overexpress Pin4-DsRed.

### FRAP

Fluorescence recovery after photobleaching (FRAP) was performed on a Zeiss LSM510 Axiovert confocal microscope. Mothers with buds smaller than 2.5 µm in diameter were selected in GF657 where Sup35-GFP foci became larger following Pin4 overexpression. Buds were completely bleached with a 488-nm laser at 100% power. After photobleaching, single scan images were collected every 5s with 3% laser and 5× zoom power. The pinhole was fully open to allow complete bleaching and to yield enough signal for fluorescent recovery.

Relative fluorescence intensity (RFI) was determined by RFI = ((Ne_t_-Ne_min_/N1_t_)/(Ne_0_-Ne_min_/N1_0_))×100, where Ne_t_ is the average intensity of the bleached bud at time t and N1_t_ is the average intensity of its non-bleached mother cell at the corresponding time used to compensate for loss in total fluorescence [95]. Ne_0_ and N1_0_ represent average intensities of the bleached bud and its non-bleached mother respectively before photobleaching. Ne_min_ is the minimum fluorescence intensity of the bud seen. The half-time that indicates the speed of mobility and the plateau level of recovery were measured by curve fitting the RFI data to a one-phase exponential association alogorithm with GraphPad Prism.

### Quantification of fluorescence intensity

Fluorescent images were acquired using the same exposure time for all the samples. Using ImageJ, cytoplasmic regions devoid of aggregates and the vacuole were selected with the “brush” selection tool. The mean fluorescence intensity in the selected area was quantified using the “measure” function. The area around the cell was selected as the background. The data for each cell was obtained by calculating the mean fluorescence in the cytoplasm subtracted by that in the background.

### α-factor arrest

Cell growth was arrested by the addition of 50 µM of the yeast mating pheromone α-factor. α-factor peptide (Trp-His-Trp-Leu-Gln-Leu-Lys-Pro-Gly-Gln-Pro-Met-Tyr) was from GenScript.

### Preparation and analysis of yeast cell lysates

Cells overexpressing *PIN4* or *PIN4-DsRED* were grown in 150 ml of 2% raffinose +2% galactose media to an A_600_ OD of 0.3–0.8, where 80% of cells contained larger Sup35-GFP foci. Lysates were prepared as described [32]. For chaperone analysis, equal amounts of total proteins in precleard lysates were analyzed by Western blot using previously described antibodies [38]. Monoclonal anti-Pgk1 antibody was from Invitrogen. For semi-denaturing detergent agarose gel electrophoresis (SDD-AGE), 50–80 µg of total protein in precleared lysates were incubated for 7 min in sample buffer with 2% SDS at room temperature and resolved on 1.5% agarose gels [96].

### Immunocapture of cell lysates on magnetic beads

Immunocapture experiments were essentially as described [38] with the following changes: 750 µl of a higher salt lysis buffer [LB2: 40 mM Tris-HCl (pH 7.5), 150 mM KCl, 5 mM MgCl_2_, 10% glycerol] was used; 500 µl lysates of 0.5–1.0 mg/ml proteins were incubated with 3 µl of α-DsRed antibody for 2 hrs on ice; samples were mixed with 50 µl magnetic beads with immobilized G protein (Miltenyi Biotec) and incubated on ice for 30 min. Finally, beads were washed with 1.0 ml of each of the following solutions at 4°C in the following order to remove nonspecifically bound proteins: LB2 with 1% Triton X-100; LB2, 210 mM KCl, 1% Triton X-100; LB2 with 1% Triton X-100; LB2; LB1 [40 mM Tris-HCl (pH 7.5), 50 mM KCl, 5 mM MgCl_2_, 5% glycerol]; 20 mM Tris-HCl (pH 7.6). α-DsRed for immunocapturing was monoclonal antibody from Clontech, and α-DsRed for detection was a polyclonal antibody from Santa Cruz Biotechnology.

## Supporting Information

Figure S1 There is no growth advantage of [*psi* ^−^] over [*PSI* ^+^] cells upon overexpression of Q/N-rich domains. Amplification of high copy plasmids encoding Q/N-rich domains exhibits no differences in cell viability in the absence or presence of [*PSI* ^+^]. Isogenic [*PIN* ^+^] strains lacking (−) [*PSI* ^+^] (L1749) or containing (+) weak [*PSI* ^+^] (L1758) were each transformed with the high copy (*URA3*, *leu2-d*) plasmids with an insert encoding the indicated Q/N-rich domain, or the control empty vector pHR81. Two transformants for each plasmid were 10-fold serially diluted and spotted on SD-Leu to amplify library plasmids (left panels), and SD-Ura to maintain plasmid low copy number (right panels). One representative transformant for each plasmid was photographed after 4 days of incubation.
(TIF)

Figure S2 Cells with larger Sup35-GFP aggregates caused by overexpressed Pin4C are capable of propagating [*PSI* ^+^]. Representative fluorescent images of strong [*PSI* ^+^][*PIN* ^+^] cells expressing the chromosomal *SUP35-GFP* fusion (GF657) after overexpressing Pin4C. Images were taken after inducing pHR81*GAL*-*PIN4C* overnight, when ∼80% cells in the culture contained larger Sup35-GFP foci (top, left panel) and after continued induction in presence of excess Pin4C for another 4 days (top, right panel). Following the imaging, cells were plated onto rich media (YPD) to determine the prion state (bottom panels). Colonies on the right are smaller because the plate was more crowded.
(TIF)

Figure S3 Sup35-GFP foci reduce in number progressively in dividing cells. Single strong [*PSI* ^+^][*PIN* ^+^] *SUP35-GFP* expressing (GF657) cells carrying pHR81*GAL*-*PIN4C-DsRED* were micromanipulated and grown on 2% raffinose + 2% galactose to induce Pin4C-DsRed for ∼24 hrs. A portion of the microcolony is shown as a GFP image. Sup35-GFP foci increased in size and were reduced in number progressively in cells dividing from the center to the edge of the microcolony. Single huge faint fluorescent areas in some cells are due to leakage of Pin4C-Dsred foci into the GFP channel; such foci were never observed in the GFP channel when overexpressing the Pin4C not tagged with DsRed (see Figure 2A).
(TIF)

Figure S4 The Hsp104 level is slightly reduced following Pin4C overexpression. (A) Overexpression of Pin4C has no notable effect on the expression levels of chaperones. Lysates of strong [*PSI* ^+^][*PIN* ^+^] (GF657) following overnight overexpression of Pin4C from pHR81*GAL-PIN4C* were analyzed by immunoblotting with the indicated antibodies. Ribosomal protein L3 was used as an internal loading control. Control cultures were transformed with the pHR81*GAL* vector. (B) Hsp104 expression was visualized using a PhosphorImager scanning system after immunoblotting the lysates described above with anti-Hsp104 antibody and also with anti-Pgk1 antibody. (C) A heterozygous disruption of *HSP104* has no effect on [*PSI* ^+^] propagation. Genomic *SUP35-GFP* strong [*PSI* ^+^][*PIN* ^+^] (GF845) carrying pHR81*GAL*-*PIN4C* (↑Pin4C, *HSP104*), or diploids from a cross of GF845 harboring the empty vector pHR81*GAL* to a [*psi* ^−^] strain with a disruption of *HSP104* and genomic *SUP35-GFP* (GF844) harboring the empty vector pRS413 (↑vectors, *HSP104/Δ*), were grown on plasmid selective glucose medium, and replica-plated onto plasmid selective galactose to induce the *GAL* promoter, and then 10-fold serial diluted (10^5^∼10^0^ cells from left to right) and spotted onto YPD glucose medium. Shown is a representative image. There were no red colonies indicative of [*psi^−^*] observed in the *HSP104* heterozygous disruption background.
(TIF)

Figure S5 There is no difference in cell growth with overexpressed Pin4C in the presence and absence of excess Hsp104^T160M^. Strong [*PSI* ^+^][*PIN* ^+^] *SUP35-GFP* cells (GF657) with pHR81*GAL*-*PIN4C* and pRS413*GAL*-*HSP104^T160M^* (↑Pin4C, ↑Hsp104^T160M^); or with pHR81*GAL*-*PIN4C* and empty vector pRS413*GAL* (↑Pin4C); or with pRS413*GAL*-*HSP104^T160M^* and pHR81*GAL* (↑Hsp104^T160M^); or with both empty vectors pHR81*GAL* and pRS413*GAL* (↑vectors) were grown on plasmid selective glucose medium, and then 10-fold serially diluted (10^4^∼10^2^ cells from left to right) and spotted onto plasmid selective galactose to induce the *GAL* promoter. Transformants spotted onto plasmid selective glucose medium (-Ura-Trp) were used as a control.
(TIF)

Figure S6 Effects of overexpression of Cyc8C or Pin3 on chaperone levels. (A) Overexpression of Pin3 caused a slight increase in the levels of Hsp104, Sse1, Sis1 and Ydj1. Lysates of cells with GFP tagged endogenous Hsp104 following overnight overexpression of Pin3 from pHR81*GAL-PIN3* were analyzed by stripping and immunostaining the same blot with the indicated antibodies, except that another bolt was immunostained with anti-Ydj1 and anti-Pgk1. Pgk1 was used as an internal loading control. Control cultures were transformed with the pHR81*GAL* vector. (B) Overexpressed Cyc8C caused a dramatic increase in Hsp104 levels. Lysates of Hsp104-GFP cells following overnight overexpression of Cyc8C or the empty vector pHR81 were analyzed by immunoblotting with the indicated antibodies. Pgk1 was used as an internal loading control.
(TIF)

Table S1 Quantification of Hsp104 levels upon Pin4C overexpression. The Hsp104 level was quantified using ImageQuant software and normalizing against the internal Pgk1 control. The normalized Hsp104 level in cells overexpressing Pin4C was compared with that in cells with the empty vector. Data was presented as mean ± SD, n = 5.
(DOC)
