# Supplementary figures and images for: Heterologous Gln/Asn-Rich Proteins Impede the Propagation of Yeast Prions by Altering Chaperone Availability
Source: PLoS Genet. 2013 Jan 24;9(1):e1003236. doi: 10.1371/journal.pgen.1003236 (PMC3554615; doi:10.1371/journal.pgen.1003236)

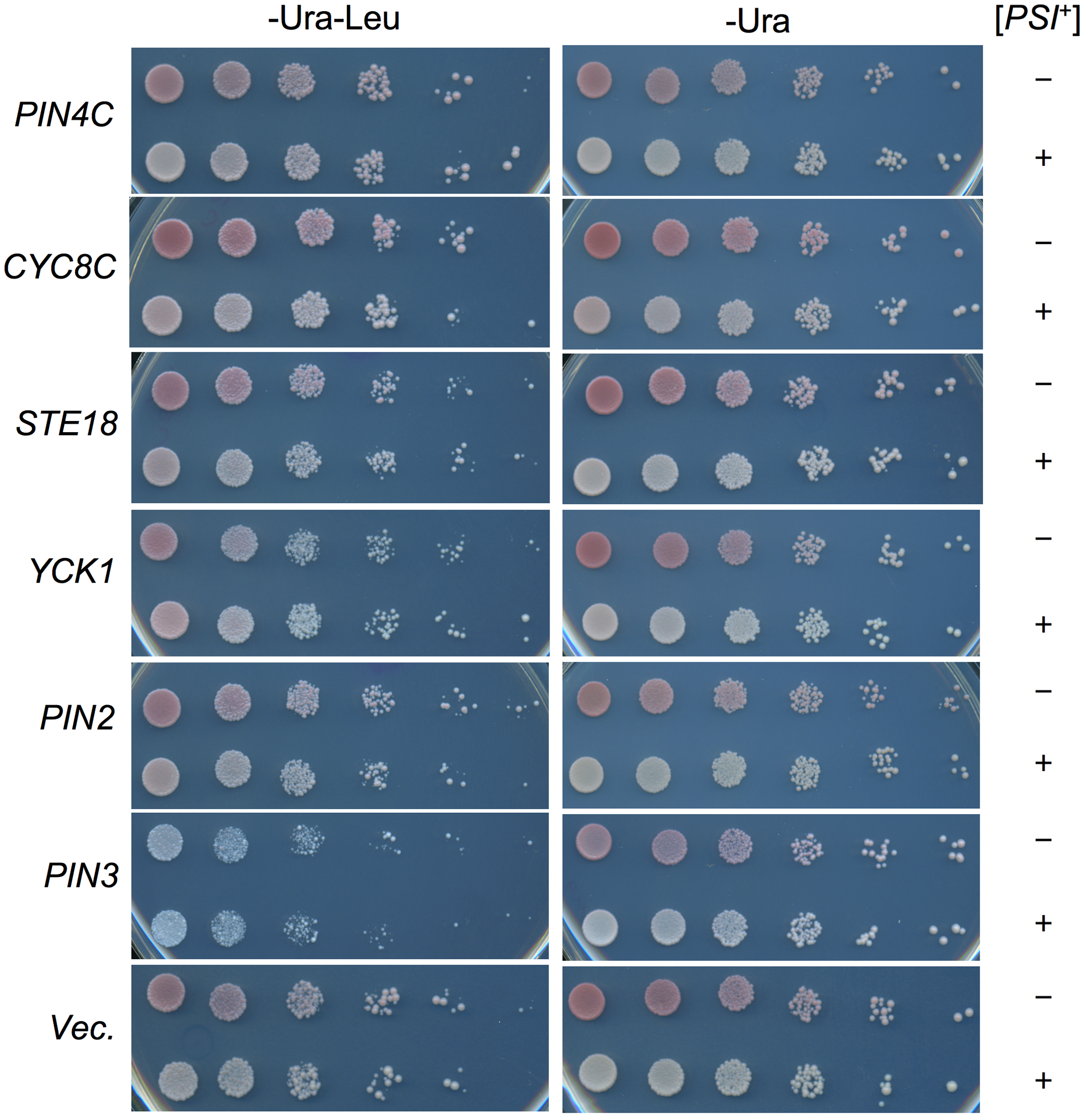

Supplement: Figure S1 — There is no growth advantage of [psi −] over [PSI +] cells upon overexpression of Q/N-rich domains. Amplification of high copy plasmids encoding Q/N-rich domains exhibits no differences in cell viability in the absence or presence of [PSI +]. Isogenic [PIN +] strains lacking (−) [PSI +] (L1749) or containing (+) weak [PSI +] (L1758) were each transformed with the high copy (URA3, leu2-d) plasmids with an insert encoding the indicated Q/N-rich domain, or the control empty vector pHR81. Two transformants for each plasmid were 10-fold serially diluted and spotted on SD-Leu to amplify library plasmids (left panels), and SD-Ura to maintain plasmid low copy number (right panels). One representative transformant for each plasmid was photographed after 4 days of incubation. (TIF) [file pgen.1003236.s001.tif]

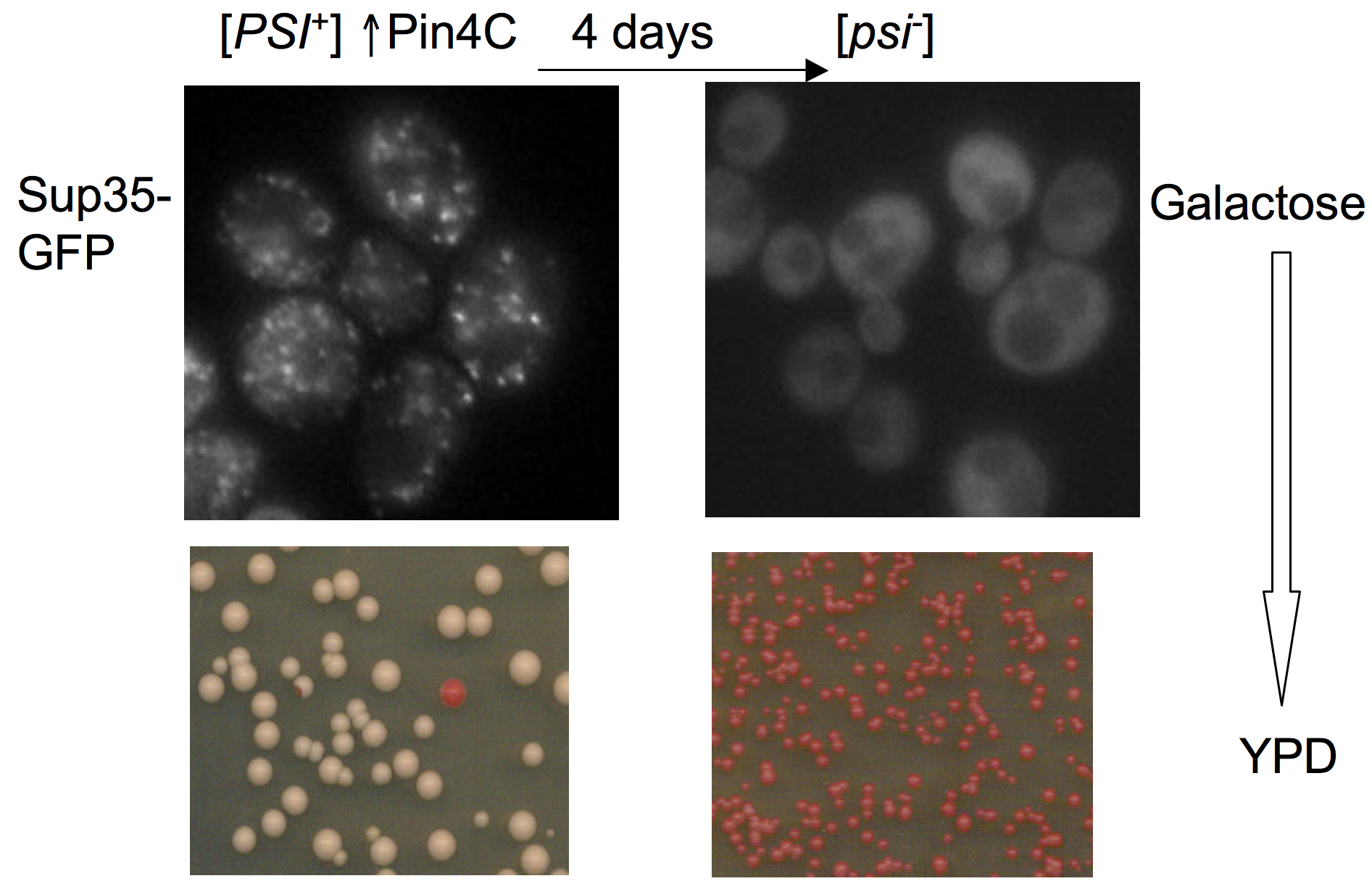

Supplement: Figure S2 — Cells with larger Sup35-GFP aggregates caused by overexpressed Pin4C are capable of propagating [PSI +]. Representative fluorescent images of strong [PSI +][PIN +] cells expressing the chromosomal SUP35-GFP fusion (GF657) after overexpressing Pin4C. Images were taken after inducing pHR81GAL-PIN4C overnight, when ∼80% cells in the culture contained larger Sup35-GFP foci (top, left panel) and after continued induction in presence of excess Pin4C for another 4 days (top, right panel). Following the imaging, cells were plated onto rich media (YPD) to determine the prion state (bottom panels). Colonies on the right are smaller because the plate was more crowded. (TIF) [file pgen.1003236.s002.tif]

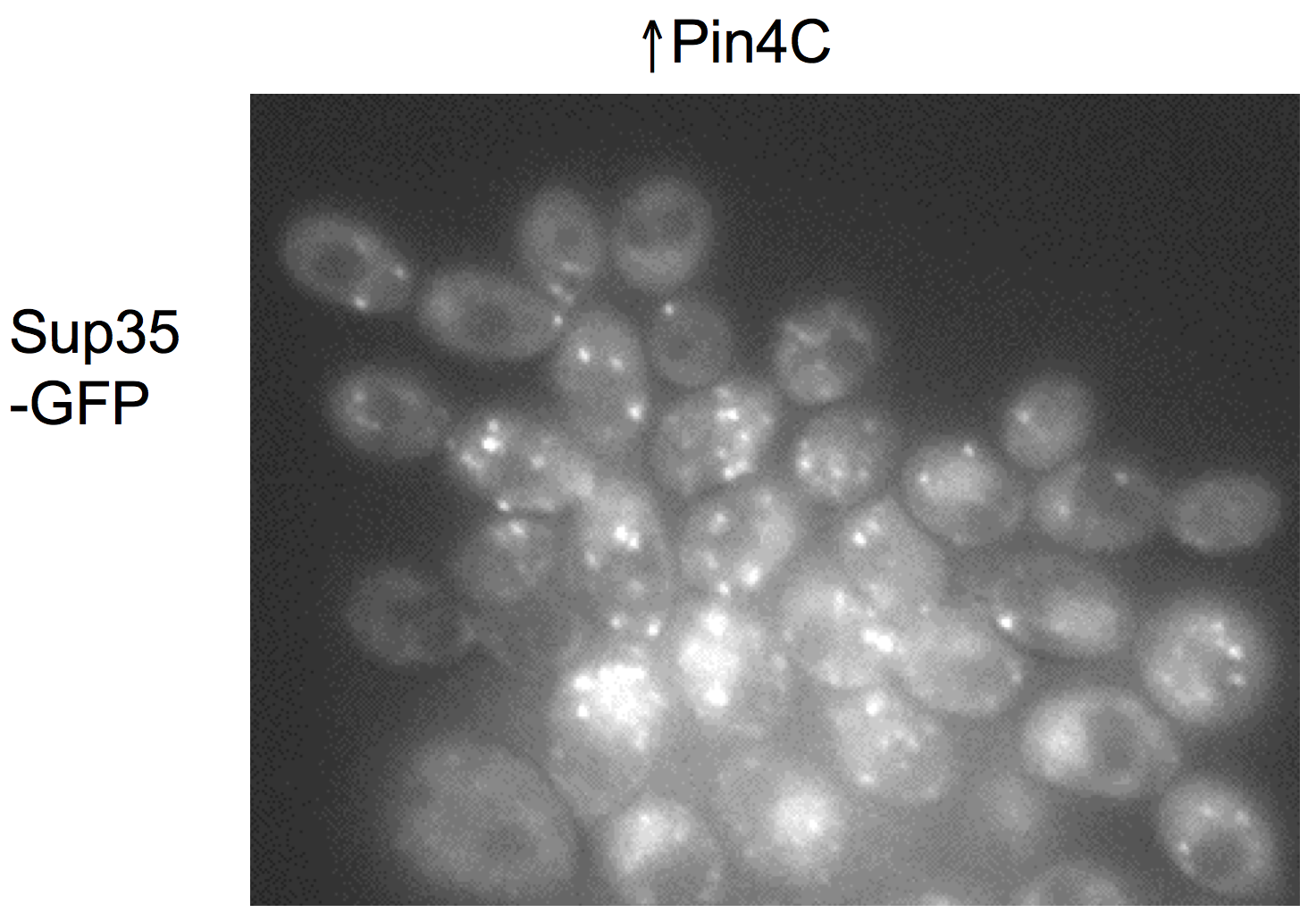

Supplement: Figure S3 — Sup35-GFP foci reduce in number progressively in dividing cells. Single strong [PSI +][PIN +] SUP35-GFP expressing (GF657) cells carrying pHR81GAL-PIN4C-DsRED were micromanipulated and grown on 2% raffinose + 2% galactose to induce Pin4C-DsRed for ∼24 hrs. A portion of the microcolony is shown as a GFP image. Sup35-GFP foci increased in size and were reduced in number progressively in cells dividing from the center to the edge of the microcolony. Single huge faint fluorescent areas in some cells are due to leakage of Pin4C-Dsred foci into the GFP channel; such foci were never observed in the GFP channel when overexpressing the Pin4C not tagged with DsRed (see Figure 2A). (TIF) [file pgen.1003236.s003.tif]

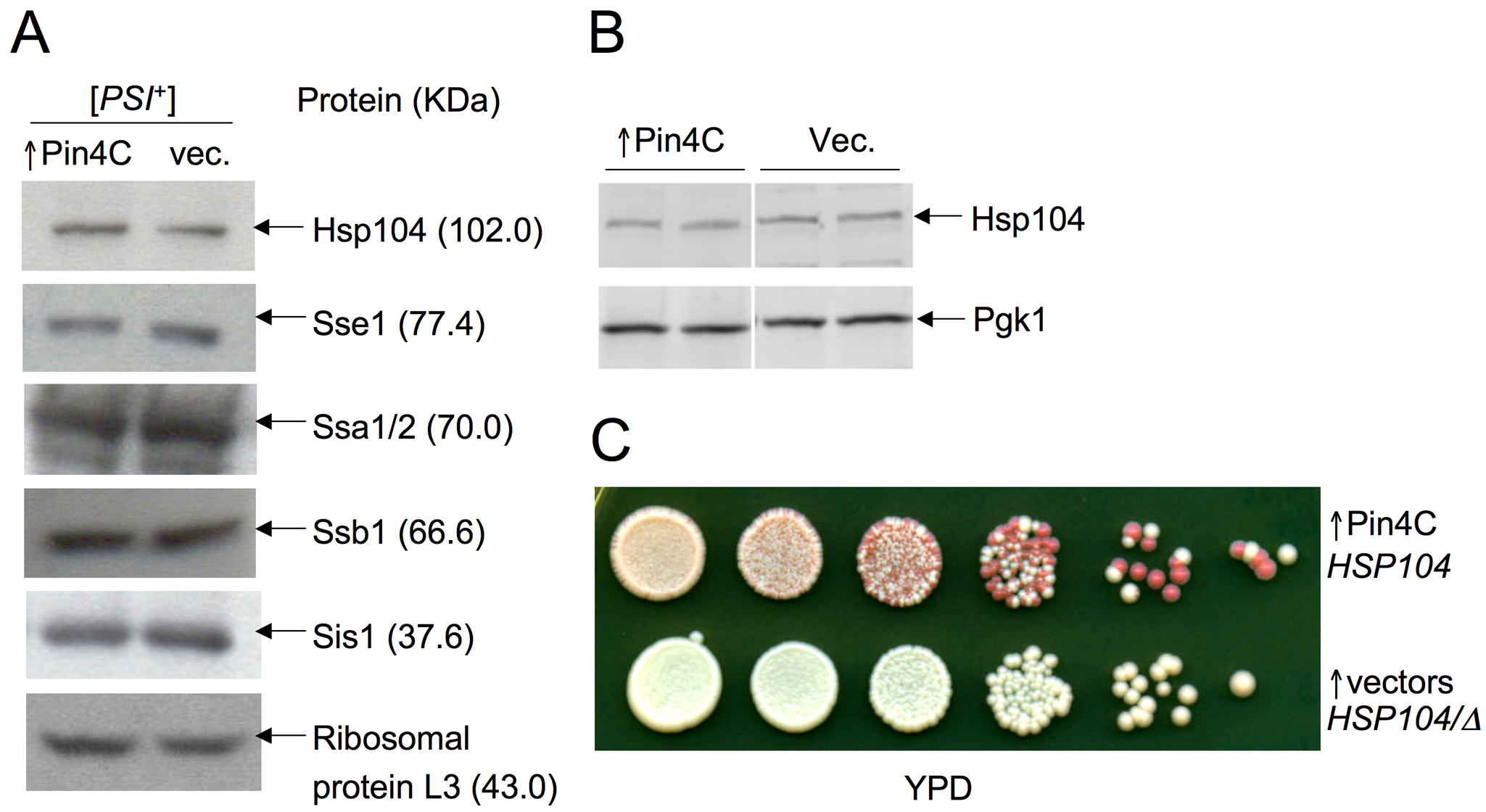

Supplement: Figure S4 — The Hsp104 level is slightly reduced following Pin4C overexpression. (A) Overexpression of Pin4C has no notable effect on the expression levels of chaperones. Lysates of strong [PSI +][PIN +] (GF657) following overnight overexpression of Pin4C from pHR81GAL-PIN4C were analyzed by immunoblotting with the indicated antibodies. Ribosomal protein L3 was used as an internal loading control. Control cultures were transformed with the pHR81GAL vector. (B) Hsp104 expression was visualized using a PhosphorImager scanning system after immunoblotting the lysates described above with anti-Hsp104 antibody and also with anti-Pgk1 antibody. (C) A heterozygous disruption of HSP104 has no effect on [PSI +] propagation. Genomic SUP35-GFP strong [PSI +][PIN +] (GF845) carrying pHR81GAL-PIN4C (↑Pin4C, HSP104), or diploids from a cross of GF845 harboring the empty vector pHR81GAL to a [psi −] strain with a disruption of HSP104 and genomic SUP35-GFP (GF844) harboring the empty vector pRS413 (↑vectors, HSP104/Δ), were grown on plasmid selective glucose medium, and replica-plated onto plasmid selective galactose to induce the GAL promoter, and then 10-fold serial diluted (105∼100 cells from left to right) and spotted onto YPD glucose medium. Shown is a representative image. There were no red colonies indicative of [psi−] observed in the HSP104 heterozygous disruption background. (TIF) [file pgen.1003236.s004.tif]

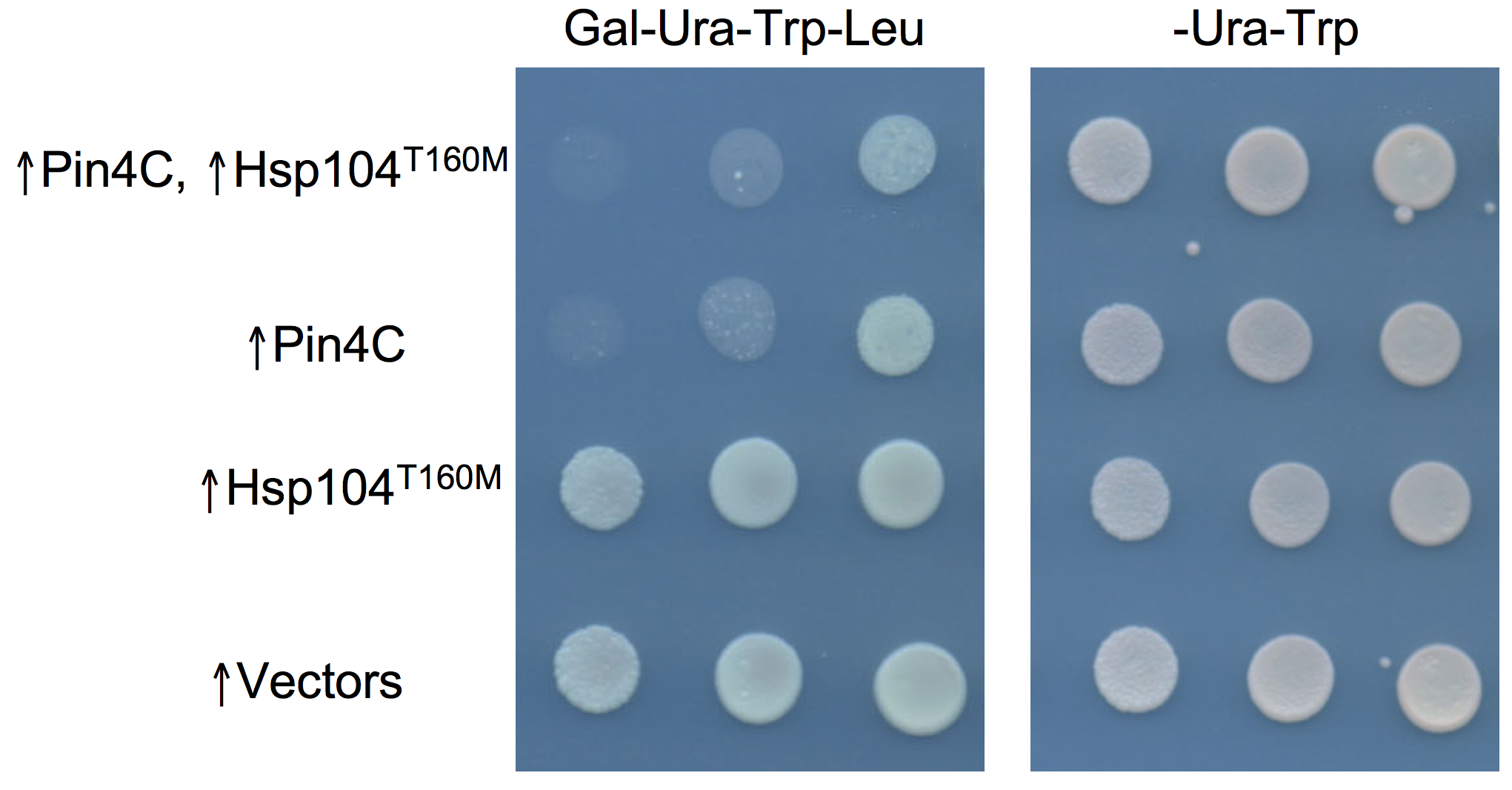

Supplement: Figure S5 — There is no difference in cell growth with overexpressed Pin4C in the presence and absence of excess Hsp104T160M. Strong [PSI +][PIN +] SUP35-GFP cells (GF657) with pHR81GAL-PIN4C and pRS413GAL-HSP104T160M (↑Pin4C, ↑Hsp104T160M); or with pHR81GAL-PIN4C and empty vector pRS413GAL (↑Pin4C); or with pRS413GAL-HSP104T160M and pHR81GAL (↑Hsp104T160M); or with both empty vectors pHR81GAL and pRS413GAL (↑vectors) were grown on plasmid selective glucose medium, and then 10-fold serially diluted (104∼102 cells from left to right) and spotted onto plasmid selective galactose to induce the GAL promoter. Transformants spotted onto plasmid selective glucose medium (-Ura-Trp) were used as a control. (TIF) [file pgen.1003236.s005.tif]

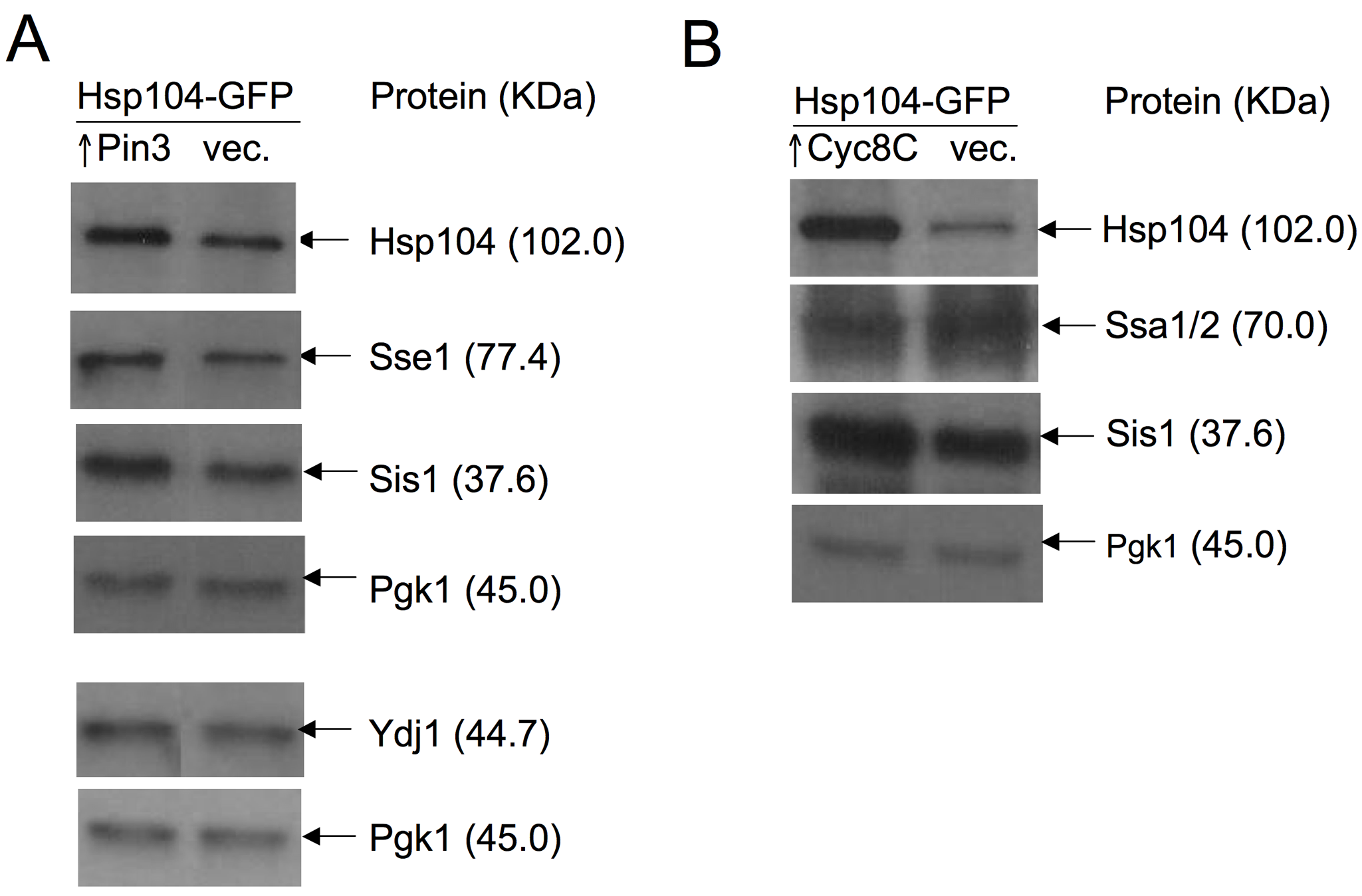

Supplement: Figure S6 — Effects of overexpression of Cyc8C or Pin3 on chaperone levels. (A) Overexpression of Pin3 caused a slight increase in the levels of Hsp104, Sse1, Sis1 and Ydj1. Lysates of cells with GFP tagged endogenous Hsp104 following overnight overexpression of Pin3 from pHR81GAL-PIN3 were analyzed by stripping and immunostaining the same blot with the indicated antibodies, except that another bolt was immunostained with anti-Ydj1 and anti-Pgk1. Pgk1 was used as an internal loading control. Control cultures were transformed with the pHR81GAL vector. (B) Overexpressed Cyc8C caused a dramatic increase in Hsp104 levels. Lysates of Hsp104-GFP cells following overnight overexpression of Cyc8C or the empty vector pHR81 were analyzed by immunoblotting with the indicated antibodies. Pgk1 was used as an internal loading control. (TIF) [file pgen.1003236.s006.tif]
