# Supplementary material for: Heterologous Gln/Asn-Rich Proteins Impede the Propagation of Yeast Prions by Altering Chaperone Availability
Source: PLoS Genet. 2013 Jan 24;9(1):e1003236. doi: 10.1371/journal.pgen.1003236 (PMC3554615; doi:10.1371/journal.pgen.1003236)
Supplement: Table S1 — Quantification of Hsp104 levels upon Pin4C overexpression. The Hsp104 level was quantified using ImageQuant software and normalizing against the internal Pgk1 control. The normalized Hsp104 level in cells overexpressing Pin4C was compared with that in cells with the empty vector. Data was presented as mean ± SD, n = 5. (DOC) [file pgen.1003236.s007.doc]

**Table S1. Quantification of Hsp104 levels upon Pin4C overexpression.**

| Hsp104  /Pgk1 | 1 | 2 | 3 | 4 | 5 |
| --- | --- | --- | --- | --- | --- |
| ↑Pin4C | 0.32 | 0.24 | 1.40 | 0.6 | 0.62 |
| ↑Vec. | 0.38 | 0.36 | 1.99 | 0.59 | 0.66 |
| ↑Pin4C/vec. | 0.84 | 0.67 | 0.70 | 1.02 | 0.94 |
| Mean | 0.83±0.15 | | | | |
